# Promotion of behavior and neuronal function by reactive oxygen species in *C*. *elegans*

**DOI:** 10.1038/ncomms13234

**Published:** 2016-11-08

**Authors:** Guang Li, Jianke Gong, Haoyun Lei, Jianfeng Liu, X. Z. Shawn Xu

**Affiliations:** 1 College of Life Science and Technology, Collective Innovation Center for Brain Science and Key Laboratory of Molecular Biophysics of MOE, Huazhong University of Science and Technology, Wuhan, Hubei 430074, China; 2 Life Sciences Institute, University of Michigan, Ann Arbor, Michigan 48109, USA; 3 Department of Molecular and Integrative Physiology, University of Michigan Medical School, Ann Arbor, Michigan 48109, USA

## Abstract

Reactive oxygen species (ROS) are well known to elicit a plethora of detrimental effects on cellular functions by causing damages to proteins, lipids and nucleic acids. Neurons are particularly vulnerable to ROS, and nearly all forms of neurodegenerative diseases are associated with oxidative stress. Here, we report the surprising finding that exposing *C. elegans* to low doses of H_2_O_2_ promotes, rather than compromises, sensory behavior and the function of sensory neurons such as ASH. This beneficial effect of H_2_O_2_ is mediated by an evolutionarily conserved peroxiredoxin-p38/MAPK signaling cascade. We further show that p38/MAPK signals to AKT and the TRPV channel OSM-9, a sensory channel in ASH neurons. AKT phosphorylates OSM-9, and such phosphorylation is required for H_2_O_2_-induced potentiation of sensory behavior and ASH neuron function. Our results uncover a beneficial effect of ROS on neurons, revealing unexpected complexity of the action of oxidative stressors in the nervous system.

## Abstract (web summary)

The deleterious role of reactive oxygen species has been widely reported in the nervous system. Here the authors report that surprisingly, low doses of H_2_O_2_ in fact enhances sensory neuron function and promotes sensory behaviors in *C. elegans*.

## Graphical abstract

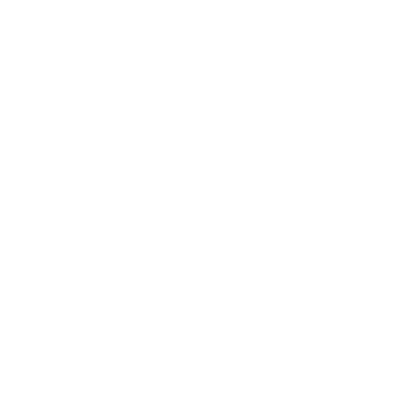

Reactive oxygen species (ROS) cause a wide range of detrimental effects at the cellular level, leading to protein aggregation, aberrant cell signaling, and ultimately cell senescence and death^1^,^2^. They do so by oxidizing cellular components such as proteins, lipids and nucleic acids, as well as via cellular signaling^1^,^3^. Exposure to ROS also triggers defense mechanisms in the cell by up-regulating the production of antioxidants that remove ROS (ref. 2). An imbalance between ROS production and removal results in oxidative stress^2^. Neurons are among the cell types that are most vulnerable to oxidative insults, largely due to their high metabolic rate and low regenerative potential^2^,^4^. The deleterious role of ROS in the nervous system has been well documented. For example, the progression of neurodegenerative diseases, such as Alzheimer's disease, Parkinson's disease and amyotrophic lateral sclerosis, is associated with oxidative stress^2^,^4^. Ischemia, seizure, and traumatic brain injury all trigger excess production of ROS, which contribute to the brain damages caused by these pathophysiological conditions^2^,^4^.

Notwithstanding the detrimental impact of ROS, these agents are produced in cells under physiological conditions, such as mitochondrial respiration and innate immune response, and regulate cellular signaling^3^,^5^. For example, an increase in the production of ROS by mitochondria may induce adaptive responses and subsequently potentiate stress resistance in animals, a phenomenon called mitohormesis^6^,^7^. This led us to question if ROS have other types of effects on brain function.

Here, we explore this possibility in the nematode *Caenorhabditis elegans*, a popular genetic model organism with a nervous system that has been very well characterized. We find that while high concentrations of H_2_O_2_ are detrimental to *C. elegans*, low doses of H_2_O_2_, surprisingly, promote sensory behavior and also potentiate sensory neuronal function. Reducing the level of endogenous H_2_O_2_ produces an opposite effect, suggesting that the effect of H_2_O_2_ is physiological. The beneficial role of H_2_O_2_ is mediated by an evolutionarily-conserved peroxiredoxin-p38/MAPK signaling axis that signals to AKT and the sensory channel TRPV/OSM-9. We further show that AKT regulates TRPV/OSM-9 sensory channel through phosphorylation. These findings reveal a beneficial effect of ROS on nervous system function.

## Results

### Low doses of H_2_O_2_ promote sensory behavior

**Figure 1:**
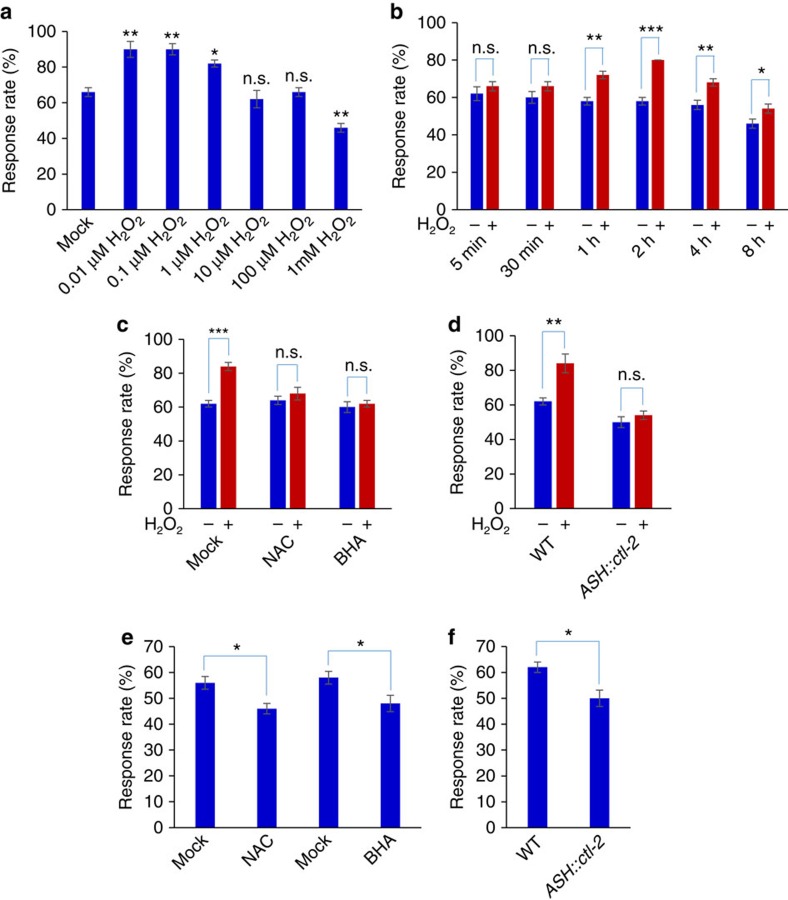
Low doses of H_2_O_2_ promote osmotic avoidance behavior. (**a**) Low doses of H_2_O_2_ promote osmotic avoidance behavior while high concentrations of H_2_O_2_ inhibit it. Worms were pre-incubated with varying concentrations of H_2_O_2_ for 2 h. After a brief recovery on seeded NGM plates (up to 2 h), worms were tested for avoidance response to glycerol. To avoid a ceiling effect which would mask behavioral potentiation, a non-saturating concentration of glycerol (0.5 M) was used to challenge the worm. *n*≥20; *P<0.05, **P<0.005 (ANOVA with Dunnett's test); Error bars: s.e.m. (**b**) Exposing worms to H_2_O_2_ for one hour is sufficient to induce behavioral potentiation. Worms were pre-treated with 0.1 μM of H_2_O_2_ for varying durations of time. *n*≥20; *P<0.05, **P<0.005, ***P<0.0005 (*t* test); Error bars: s.e.m. (**c**) The antioxidants NAC and BHA block H_2_O_2_-induced behavioral potentiation. BHA (butylated hydroxyanisole, 25 μM) and NAC (N-acetyl-cysteine, 1 mM) were included during H_2_O_2_ (0.1 μM ) treatment. *n*≥10; ***P<0.0005 (ANOVA); Error bars: s.e.m. (**d**) Transgenic expression of the catalase gene *ctl-2* in ASH neurons blocks H_2_O_2_-induced behavioral potentiation. H_2_O_2_: 0.1 μM. The *sra-6* promoter was used to drive expression of *ctl-2* in ASH neurons. *n*≥10; **P<0.005 (ANOVA); Error bars: s.e.m. (**e**) Reducing the level of endogenous H_2_O_2_ suppresses osmotic avoidance behavior. Worms were treated with BHA or NAC for 2 h, and right after the treatment, they were assayed for osmotic avoidance behavior. *n*≥10; *P<0.05 (ANOVA); Error bars: s.e.m. (**f**) Reducing the level of endogenous H_2_O_2_ in ASH neurons suppresses osmotic avoidance behavior. Transgenic expression of the catalase gene *ctl-2* in ASH neurons suppressed osmotic avoidance behavior. H_2_O_2_: 0.1 μM. *n*≥10; *P<0.05 (ANOVA); Error bars: s.e.m. Note: 0.1 μM H_2_O_2_ was used to induce potentiation of osmotic avoidance behavior and ASH neuron sensory response throughout the paper unless otherwise indicated.

Despite its small size, *C. elegans* nervous system drives a rich repertoire of behaviors, particularly sensory behaviors^8^. Previous work examining the role of ROS on worm behavior has mainly focused on high doses of H_2_O_2_, and found that exposing worms to the millimolar range of H_2_O_2_ severely compromises behavior^9^. We thus started by testing a wider range of concentrations of H_2_O_2_ (Fig. 1a). We incubated worms with varying concentrations of H_2_O_2_, and after a brief recovery, we examined their behavioral responses to sensory cues (see Materials and Methods). We focused on osmotic avoidance behavior, one of the best characterized sensory behaviors in *C. elegans* (ref. 8). Treating worms with 1 mM H_2_O_2_ suppressed osmotic avoidance behavior by decreasing their response rate to high osmolarity solution (glycerol) (Fig. 1a and Supplementary Fig. 1), consistent with the notion that oxidative insults negatively affect worm behavior^9^. Surprisingly, exposing worms to lower concentrations of H_2_O_2_ at the micromolar and sub-micromolar range potentiated osmotic avoidance behavior (Fig. 1a and Supplementary Fig. 1). Treatment with H_2_O_2_ for 1 h was sufficient to induce such behavioral potentiation (Fig. 1b). Exposing worms to the ROS-producing agent paraquat also elicited similar effects (Supplementary Fig. 2a), while heat treatment did not (Supplementary Fig. 2b). Osmotic avoidance behavior is mediated by the polymodal sensory neuron ASH which detects high osmolarity cues^10^,^11^. This neuron also mediates octanol avoidance response^12^, and we obtained similar results with octanol avoidance behavior in worms treated with H_2_O_2_ (Supplementary Fig. 2c). These experiments reveal a beneficial role of H_2_O_2_ in promoting behavioral responses in *C. elegans*.

To verify whether the observed potentiation of behavioral responses was caused by H_2_O_2_, we tested the effect of N-acetyl-cysteine (NAC) and butylated hydroxyanisole (BHA), two commonly used antioxidants^13^. Both antioxidants blocked H_2_O_2_-induced behavioral potentiation (Fig. 1c). To provide further evidence, we took a genetic approach by overexpressing catalase in worms. Catalase specifically removes H_2_O_2_ in the cell, antagonizing the action of H_2_O_2_ (ref. 14). Overexpression of the *C. elegans* catalase gene *ctl-2* as a transgene in ASH neurons, which mediate osmosensation^10^,^11^, abolished the ability of H_2_O_2_ to induce behavioral potentiation (Fig. 1d). These results provide further evidence that H_2_O_2_ treatment can potentiate osmotic avoidance behavior.

### Reducing endogenous H_2_O_2_ level suppresses sensory behavior

As H_2_O_2_ treatment can potentiate osmotic avoidance behavior, we wondered if reducing the H_2_O_2_ level suppresses this behavior. To test this, we treated worms with BHA and NAC, two commonly used H_2_O_2_ scavengers. To better evaluate the effect of BHA and NAC, we modified the protocol by assaying the behavior right after the treatment (see Methods). We found that BHA and NAC treatment suppressed osmotic avoidance behavior (Fig. 1e), suggesting that endogenous H_2_O_2_ may have a role in potentiating this sensory behavior. To interrogate the role of H_2_O_2_ in ASH neurons, we assayed worms overexpressing *ctl-2* as a transgene specifically in ASH neurons, as catalase specifically removes H_2_O_2_. We found that this transgene suppressed osmotic avoidance behavior (Fig. 1f). These results provide further evidence supporting a role of H_2_O_2_ in promoting osmotic avoidance behavior.

### H_2_O_2_ promotes sensory neuron function

**Figure 2:**
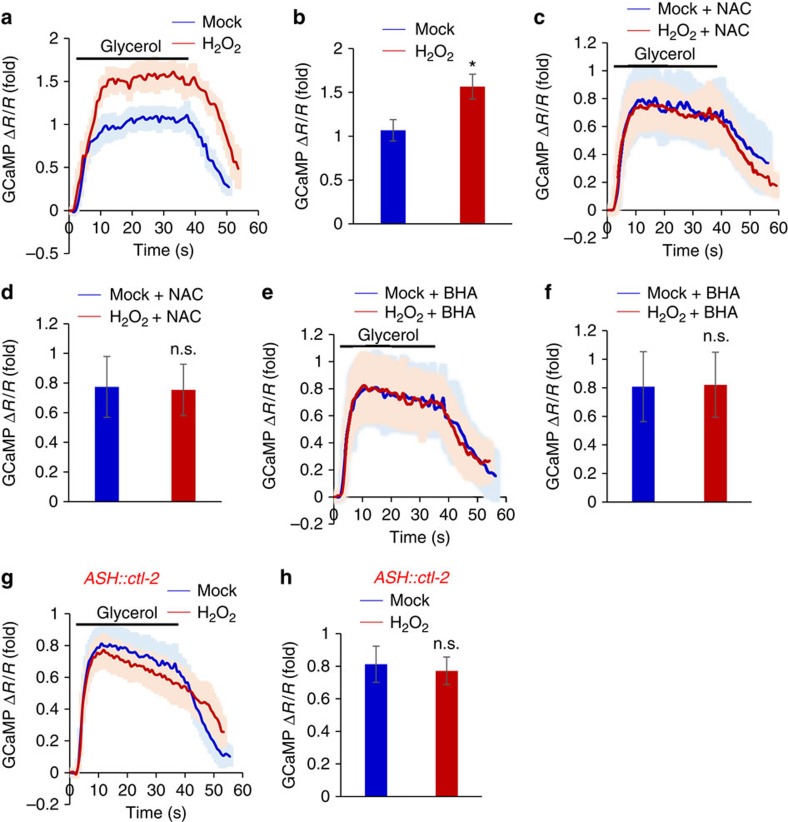
H_2_O_2_ treatment promotes ASH neuron function. (**a**,**b**) H_2_O_2_ treatment potentiates the sensory response of ASH neurons. To enable ratiometric calcium imaging, GCaMP6 and mCherry were co-expressed in ASH neurons as a transgene using the *sra-6* promoter. Worms were pre-treated with H_2_O_2_ (0.1 μM) for 2 h, and ASH neurons were recorded for their response to glycerol (0.5 M). Shades along the calcium traces in (**a**) represent error bars (s.e.m.). Bar graph in (**b**) summarizes the data in (**a**). *n*≥18; *P<0.05 (ANOVA); Error bars: s.e.m. (**c**–**f**) The antioxidants NAC and BHA block H_2_O_2_-induced potentiation of ASH sensory response. NAC or BHA was included during H_2_O_2_ treatment. Calcium imaging of ASH neurons was performed as described in (**a**). Shades along the traces in (**c**) and (**e**) represent error bars (s.e.m.). Bar graph in (**d**) and (**f**) summarizes the data in (**c**) and (**e**), respectively. *n*≥8 (ANOVA); Error bars: s.e.m. (**g**,**h**) Transgenic expression of the catalase gene *ctl-2* blocks H_2_O_2_-induced potentiation of ASH sensory response. The *sra-6* promoter was used to drive expression of *ctl-2* in ASH neurons. Calcium imaging of ASH neurons was performed as described in (**a**). Shades along the traces in (**g**) represent error bars (s.e.m.). Bar graph in (**h**) summarizes the data in (**g**). *n*≥9 (ANOVA); Error bars: s.e.m.

To investigate the neuronal mechanisms underlying H_2_O_2_-induced behavioral potentiation, we examined the function of ASH sensory neurons^10^,^11^. Calcium imaging experiments showed that H_2_O_2_ treatment potentiated the response of ASH neurons to high osmolarity solution (glycerol) (Fig. 2a,b). This potentiation effect was abolished by the antioxidants NAC and BHA (Fig. 2c–f), as well as by a transgene overexpressing the catalase gene *ctl-2* in ASH neurons (Fig. 2g,h). These data indicate that exposure to H_2_O_2_ promoted ASH neuron function, providing a neuronal basis underlying H_2_O_2_-induced behavioral potentiation.

We also examined other types of sensory behaviors such as chemotaxis. Similarly, exposing worms to low doses of H_2_O_2_ potentiated their attractive behavioral responses to odors (that is diacetyl), indicating that H_2_O_2_ treatment promoted olfactory behavior (Supplementary Fig. 3a). The sensory activity of AWA olfactory neurons, which detect diacetyl^15^, was also potentiated by H_2_O_2_ treatment as shown by calcium imaging (Supplementary Fig. 3b,c). These results demonstrate that H_2_O_2_ exposure can promote both avoidance and attractive behaviors. For simplicity, we decided to focus on osmotic avoidance behavior for further characterizations.

### p38/MAPK signaling mediates the potentiation effect of H_2_O_2_

**Figure 3:**
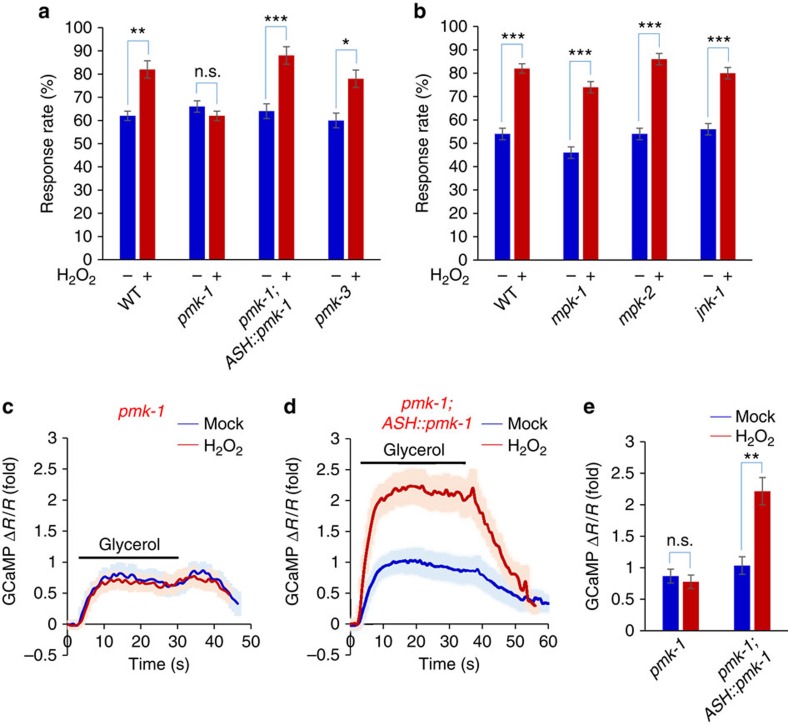
H_2_O_2_-induced potentiation of sensory behavior and ASH neuron function requires the p38/MAPK PMK-1. (**a**) H_2_O_2_-induced behavioral potentiation requires PMK-1. In *pmk-1(km25)* but not *pmk-3(ok169)* mutant worms, H_2_O_2_ treatment failed to promote osmotic avoidance behavior. This defect was rescued by a transgene expressing *pmk-1* cDNA in ASH neurons driven by the *sra-6* promoter. *n*≥20; *P<0.05, **P<0.005, ***P<0.0005 (ANOVA); Error bars: s.e.m. (**b**) H_2_O_2_-induced behavioral potentiation does not require ERK (MPK-1 and MPK-2) or JNK (JNK-1). *mpk-1(tm3476), mpk-2(tm3859)* and *jnk-1(gk7)* mutant worms were tested. *n*≥10; ***P<0.0005 (ANOVA); Error bars: s.e.m. (**c**–**e**) H_2_O_2_-induced potentiation of ASH sensory response requires PMK-1. In *pmk-1* mutant worms, H_2_O_2_ treatment can no longer promote ASH calcium response to glycerol stimulus (**c**). This defect was rescued by a transgene expressing *pmk-1* cDNA in ASH neurons driven by the *sra-6* promoter (**d**). Shades along the traces in (**c**) and (**d**) represent error bars (s.e.m.). (**e**) summarizes the data in (**c**) and (**d**). *n*≥9; **P<0.005 (ANOVA); Error bars: s.e.m.

What are the molecular mechanisms underlying H_2_O_2_-induced potentiation of sensory behavior and neuronal function? H_2_O_2_ is known to stimulate MAPK signaling^16^,^17^. The *C. elegans* genome encodes all three major classes of MAPKs: ERK, JNK and p38 (refs 18, 19). We found that loss of the p38 gene *pmk-1* abolished the ability of H_2_O_2_ to induce behavioral potentiation, while the closely related *pmk-3* was not required (Fig. 3a). In addition, mutations in ERK (*mpk-1* and *mpk-2*) and JNK (*jnk-1*) did not have a notable effect (Fig. 3b). This points to a specific role for *pmk-1* in mediating H_2_O_2_-induced behavioral potentiation, consistent with a reported role of *pmk-1* in oxidative stress signaling^20^. Expression of *pmk-1* complementary DNA (cDNA) as a transgene in ASH neurons was sufficient to rescue the phenotype (Fig. 3a), indicating that PMK-1 acts in ASH neurons. We also examined the activity of ASH by calcium imaging, and found that H_2_O_2_ treatment can no longer promote ASH sensory activity in *pmk-1* mutant worms (Fig. 3c–e), a defect that can be rescued by a transgene expressing *pmk-1* cDNA in ASH neurons (Fig. 3c–e). Thus, the p38 ortholog PMK-1 is required for H_2_O_2_-induced potentiation of sensory behavior and ASH neuron function.

**Figure 4:**
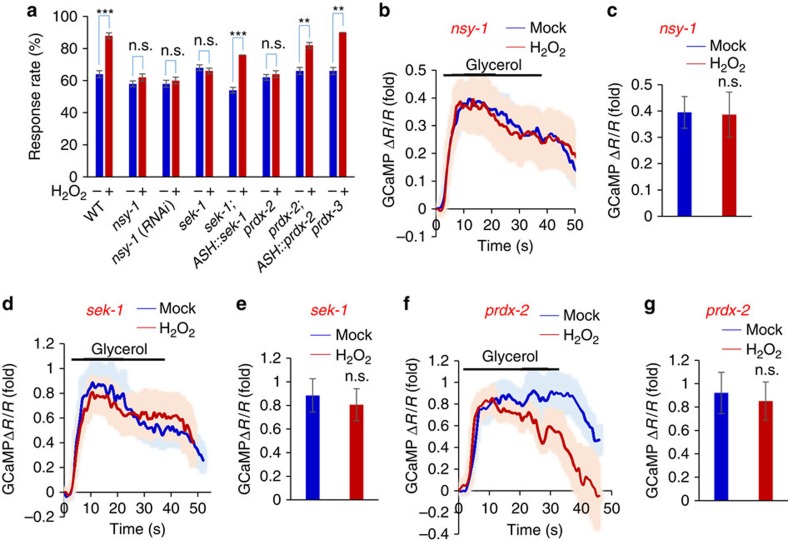
H_2_O_2_-induced potentiation of sensory behavior and ASH neuron function requires peroxiredoxin-p38/PMK-1 signaling. (**a**) H_2_O_2_-induced behavioral potentiation requires NSY-1/ASK1, SEK-1/MKK, and PRDX-2/peroxiredoxin. In *nsy-1(ok593), sek-1(km4)* and *prdx-2(gk169)* mutant worms, H_2_O_2_ treatment failed to promote osmotic avoidance behavior. RNAi of *nsy-1* gene in ASH neurons of wild-type worms recapitulated the phenotype. This was done by expressing *nsy-1* RNAi as a transgene in ASH neurons under the *sra-6* promoter. The *sek-1* and *prdx-2* mutant phenotype was rescued by a transgene expressing *sek-1* and *prdx-2* cDNA in ASH neurons driven by the *sra-6* promoter, respectively. *n*≥20; **P<0.005, ***P<0.0005 (ANOVA); Error bars: s.e.m. (**b**,**c**) H_2_O_2_-induced potentiation of ASH sensory response requires NSY-1. In *nsy-1* mutant worms, H_2_O_2_ treatment can no longer promote ASH calcium response to glycerol stimulus (**b**). Shades along the traces in (**b**) represent error bars (s.e.m.). Bar graph in (**c**) summarizes the data in (**b**). *n*≥8; Error bars: s.e.m. (**d**,**e**) H_2_O_2_-induced potentiation of ASH sensory response requires SEK-1. In *sek-1* mutant worms, H_2_O_2_ treatment can no longer promote ASH calcium response to glycerol stimulus (**d**). Shades along the traces in (**d**) represent error bars (s.e.m.). Bar graph in (**e**) summarizes the data in (**d**). *n*≥9; Error bars: s.e.m. (**f**,**g**) H_2_O_2_-induced potentiation of ASH sensory response requires PRDX-2. In *prdx-2* mutant worms, H_2_O_2_ treatment can no longer promote ASH calcium response to glycerol stimulus (**f**). Shades along the traces in (**f**) represent error bars (s.e.m.). Bar graph in (**g**) summarizes the data in (**f**). *n*≥8; Error bars: s.e.m.

p38 is activated by a conserved MAPK signaling cascade that has been extensively characterized biochemically and genetically in diverse organisms^17^,^18^,^19^,^21^. In *C. elegans*, the ASK1/MAPKKK ortholog NSY-1 and MKK/MAPKK ortholog SEK-1 are known to be in the cascade to activate p38/PMK-1 (refs 18, 19, 22, 23). We thus tested NSY-1 and SEK-1. Loss of NSY-1 blocked the ability of H_2_O_2_ to induce behavioral potentiation (Fig. 4a). RNAi of *nsy-1* gene in ASH neurons using a transgene in wild-type background also blunted H_2_O_2_-induced behavioral potentiation (Fig. 4a). Apparently, NSY-1 is required in ASH neurons to mediate behavioral potentiation. Similarly, H_2_O_2_-induced behavioral potentiation was also absent in *sek-1* mutant worms (Fig. 4a), and this phenotype can be rescued by a transgene expressing *sek-1* cDNA in ASH neurons (Fig. 4a), demonstrating a requirement for SEK-1 in ASH neurons. Further evidence came from calcium imaging experiments. In *nsy-1* mutant worms, H_2_O_2_ treatment can no longer promote ASH sensory activity (Fig. 4b,c). RNAi of *nsy-1* gene in ASH neurons using a transgene yielded a similar result (Supplementary Fig. 4a,b), suggesting that NSY-1 is required in ASH neurons to mediate neuronal potentiation. Similarly, we detected no potentiation of ASH sensory activity in *sek-1* mutant worms (Fig. 4d,e), and this phenotype was rescued by a transgene expressing *sek-1* cDNA in ASH neurons (Supplementary Fig. 4c,d). Thus, SEK-1 is also required for H_2_O_2_-induced potentiation of ASH sensory response. We conclude that the entire p38/MAPK signaling cascade is required for mediating H_2_O_2_-induced potentiation of sensory behavior and ASH neuron function.

### Role of peroxiredoxin in coupling H_2_O_2_ to p38/MAPK signaling

How is H_2_O_2_ coupled to p38/MAPK signaling? Peroxiredoxin (PRDX) came to our attention, as recent studies have unveiled an increasingly important role for these ‘antioxidants' in redox signaling^24^. In particular, 2-cys peroxiredoxin can serve as a ‘H_2_O_2_ receptor'^24^. In this case, H_2_O_2_ oxidizes a specific Cys residue in peroxiredoxin, leading to the formation of a peroxiredoxin homodimer through disulfide bonds^24^,^25^. Dimerized peroxiredoxin can then activate ASK1/MAPKKK in the p38/MAPK pathway^25^,^26^. Thus, peroxiredoxin can potentially act as a H_2_O_2_ sensor to functionally link H_2_O_2_ to p38/MAPK signaling. The worm genome encodes two 2-cys peroxiredoxin genes: *prdx-2* and *prdx-3,* and *prdx-2* has been suggested as a H_2_O_2_ sensor for lifespan regulation^25^. No defect was detected in *prdx-3* mutant worms (Fig. 4a), indicating that *prdx-3* is not required. By contrast, H_2_O_2_ treatment failed to induce behavioral potentiation in *prdx-2* mutant worms (Fig. 4a). Transgenic expression of *prdx-2* cDNA in ASH neurons rescued the mutant phenotype (Fig. 4a). Thus, PRDX-2 acts in ASH neurons to mediate H_2_O_2_-induced behavioral potentiation. In addition, calcium imaging of ASH neurons in *prdx-2* mutant worms failed to detect H_2_O_2_-induced potentiation of ASH sensory activity (Fig. 4f,g), and this defect can be rescued by a transgene expressing *prdx-2* cDNA in ASH neurons (Supplementary Fig. 4e,f). These results identify PRDX-2/peroxiredoxin as a key molecule that couples H_2_O_2_ to p38/MAPK signaling.

### AKT-1 acts downstream of p38/MAPK

Having identified a key role for the peroxiredoxin-p38/MAPK signaling pathway, we then wondered which genes act downstream of this signaling pathway to mediate H_2_O_2_-induced potentiation of sensory behavior and ASH neuron function. In redox signaling, the transcription factor Nrf2 is well known to mediate oxidative stress signaling by turning on a host of antioxidant genes to combat oxidative insults^27^. Interestingly, p38/MAPK is known to activate Nrf2 through phosphorylation^27^,^28^. We thus examined the sole *C. elegans* Nrf2 ortholog SKN-1 (ref. 29). In *skn-1* mutant worms, H_2_O_2_ lost the ability to induce behavioral potentiation (Supplementary Fig. 5a), while mutant worms lacking DAF-16/FOXO, a transcription factor also involved in oxidative stress signaling, still exhibited H_2_O_2_-induced behavioral potentiation (Supplementary Fig. 5f). This supports a role for SKN-1 in the pathway. We also found that H_2_O_2_ treatment up-regulated the expression of *gst-4* and *gcs-1* (Supplementary Fig. 5g,h), two well-characterized SKN-1 target genes^30^, suggesting that exposure to low doses of H_2_O_2_ was sufficient to trigger oxidative stress responses in worms. Much to our surprise, recording of ASH neurons by calcium imaging detected robust H_2_O_2_-induced potentiation of ASH sensory response in *skn-1* mutant worms (Supplementary Fig. 5b–e). Apparently, SKN-1 is not required for H_2_O_2_-induced potentiation of ASH neuron function. These results can be explained by a requirement of SKN-1 in those cells that act downstream of the sensory neuron ASH in the neural circuitry to drive osmotic avoidance behavior, for example, interneurons, motor neurons and/or muscles.

**Figure 5:**
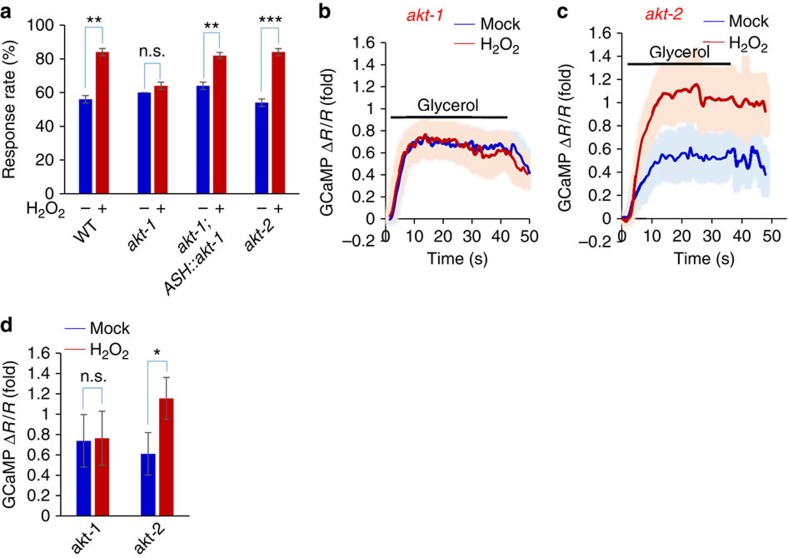
H_2_O_2_-induced potentiation of sensory behavior and ASH neuron function requires AKT-1. (**a**) H_2_O_2_-induced behavioral potentiation requires AKT-1 but not AKT-2. In *akt-1(mg306)* but not *akt-2(ok391)* mutant worms, H_2_O_2_ treatment failed to promote osmotic avoidance behavior. This phenotype was rescued by a transgene expressing *akt-1* cDNA in ASH neurons driven by the *sra-6* promoter. *n*≥20; **P<0.005, ***P<0.0005 (ANOVA); Error bars: s.e.m. (**b**–**d**) H_2_O_2_-induced potentiation of ASH sensory response requires AKT-1 but not AKT-2. In *akt-1* mutant worms, H_2_O_2_ treatment can no longer promote ASH calcium response to glycerol stimulus (**b**). No such defect was detected in *akt-2(k391)* mutant worms. Shades along the traces in (**b**) and (**c**) represent error bars (s.e.m.). Bar graph in (**d**) summarizes the data in (**b**) and (**c**). *n*≥9; Error bars: s.e.m. *P<0.05 (ANOVA).

The lack of a critical role for Nrf2/SKN-1 in ASH neurons poses a challenge to unravel the signaling events downstream of p38/PMK-1. Clearly, p38/PMK-1 substrates other than Nrf2/SKN-1 are involved. We reasoned that those proteins critical for ASH sensory activity could be potential targets of p38/PMK-1. The TRPV channel OSM-9 thus came to our attention because it is the primary sensory channel in ASH neurons^11^,^31^,^32^. However, no putative p38 phosphorylation site is predicted in OSM-9. On the other hand, five putative AKT phosphorylation sites are found in OSM-9 (see below). As it has been well established that p38/MAPK can activate AKT through a kinase complex^33^, we explored a potential involvement of AKT in mediating H_2_O_2_-induced behavioral potentiation. Two *akt* genes are encoded by the *C. elegans* genome: *akt-1* and *akt-2*. AKT-2 does not appear to be involved, as H_2_O_2_ treatment can still induce behavioral potentiation in *akt-2* mutant worms (Fig. 5a). By contrast, H_2_O_2_ treatment failed to do so in *akt-1* mutant animals (Fig. 5a). Transgenic expression of *akt-1* cDNA in ASH neurons rescued the mutant phenotype (Fig. 5a), uncovering a key role for AKT-1 in mediating H_2_O_2_-induced behavioral potentiation. Calcium imaging of ASH neurons in *akt-1* mutant worms also revealed a severe defect in H_2_O_2_-induced potentiation of ASH sensory response (Fig. 5b–d), and this phenotype can be rescued by a transgene expressing *akt-1* cDNA in ASH neurons (Supplementary Fig. 4g,h). These data identify an important role for AKT-1 in the pathway.

Taken altogether, our data suggest that PDRX-2, MAPK pathway and AKT-1 form a signaling cascade to mediate the beneficial effect of H_2_O_2_. Though the upstream-downstream relationship between these molecules has been very well characterized biochemically in diverse organisms, we sought to gather some genetic evidence to further confirm such relationship through epistatic analysis. To do so, it is important to have both gain-of-function and loss-of-function mutant alleles. Two genes in this signaling cascade have both types of alleles available: *nsy-1*/MAPKKK and *akt-1*/AKT^34^,^35^. Worms carrying gain-of-function alleles of *nsy-1* or *akt-1* showed enhanced responses to high osmolarity solution (Supplementary Fig. 6a), consistent with a role for these two genes in promoting osmotic avoidance behavior. As reducing the level of endogenous H_2_O_2_ by a transgene overexpressing the catalase CTL-2 inhibited osmotic avoidance behavior (Fig. 1f and Supplementary Fig. 6a), we tested whether *nsy-1* and *akt-1* gain-of-function alleles can suppress this inhibitory effect, and found that they did (Supplementary Fig. 6a). This is consistent with the view that MAPK pathway and AKT act downstream of H_2_O_2_. We also found that *akt-1* loss-of-function mutation suppressed the phenotype of *nsy-1* gain-of-function mutant worms (Supplementary Fig. 6b), and *vice versa* (Supplementary Fig. 6c), consistent with the notion that AKT acts downstream of MAPK pathway. These epistatic analyses lend support to our model.

### The detrimental effect of H_2_O_2_ does not require PMK-1 or AKT-1

As treating worms with high concentrations of H_2_O_2_ (for example 1 mM) suppressed osmotic avoidance behavior (Fig. 1a), we wondered if this detrimental effect of H_2_O_2_ is also mediated by the same signaling cascade. Interestingly, while H_2_O_2_-induced suppression of osmotic avoidance behavior still required the ‘H_2_O_2_ receptor' PRDX-2, it did not depend on MAPK/PMK-1 (Supplementary Fig. 7a); nor did it rely on AKT-1 (Supplementary Fig. 7a). Calcium imaging of ASH neurons yielded similar results (Supplementary Fig. 7b–i). We thus suggest that the beneficial and detrimental effects of H_2_O_2_ are likely to be mediated by different signaling pathways.

### A key role of the putative AKT site Thr10 in OSM-9

**Figure 6:**
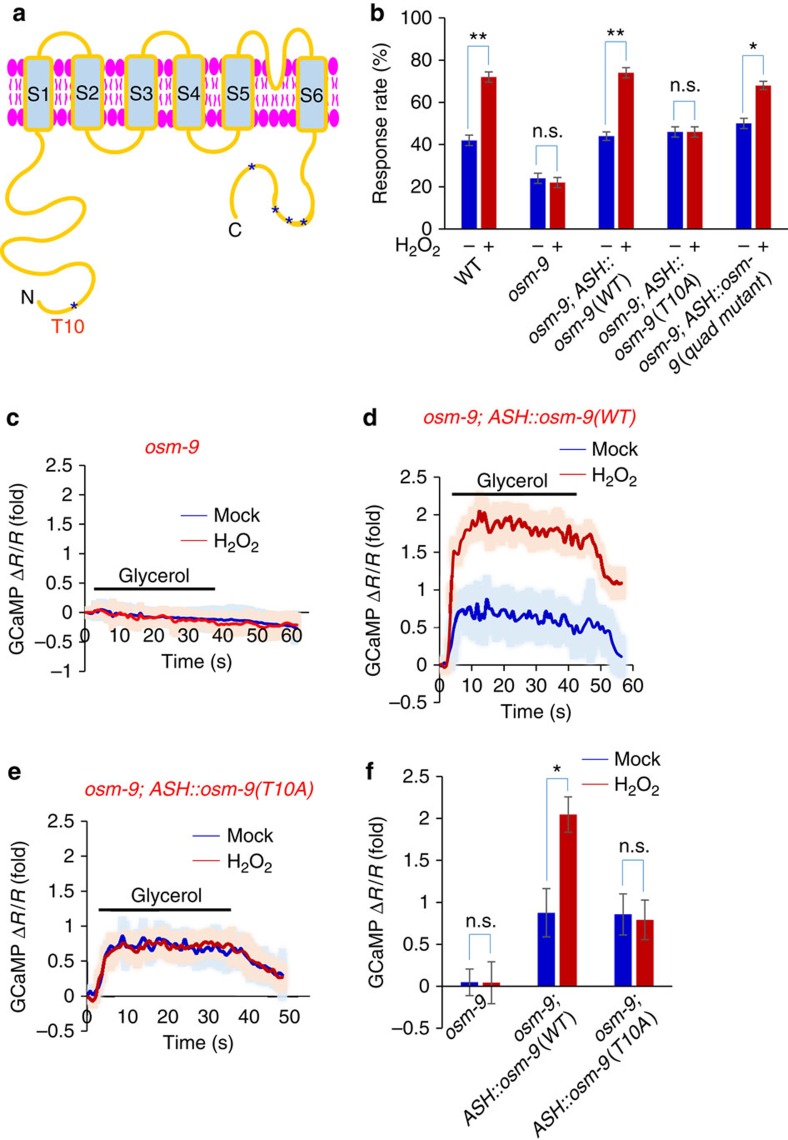
The putative AKT phosphorylation site T10 in OSM-9 is required for H_2_O_2_-induced potentiation of sensory behavior and ASH function. (**a**) Schematic of OSM-9 domain organization. Both the N and C-terminal fragments of OSM-9 are located intracellularly. Asterisks denote the putative AKT phosphorylation sites, with one at the N-terminus (T10) and the other four at the C-terminus (T769, T771, T787 and S839). These sites are predicted by Scansite. The N-terminal point mutation T10A is indicated. (**b**) The putative AKT phosphorylation site T10 in OSM-9 is required for H_2_O_2_-induced behavioral potentiation. *osm-9(ky10)* mutants failed to respond to glycerol stimulus. The basal response rate in the mutant arose from spontaneous reversal events that are present in the absence of glycerol stimulation. Wild-type and mutant forms of OSM-9 were expressed as transgenes in ASH neurons under the *sra-6* promoter. Wild-type *osm-9* transgene (*ASH::osm-9*), as well as *osm-9* transgene with all the four putative C-terminal AKT sites mutated (that is *ASH::osm-9(quad mutant)*) rescued both the basal and H_2_O_2_-induced potentiation of osmotic avoidance response. However, *osm-9* transgene harboring the point mutation T10A (that is *ASH::osm-9(T10A)*) failed to rescue H_2_O_2_-induced potentiation of osmotic avoidance response, though it rescued the basal avoidance behavior. *n*≥10; *P<0.05, **P<0.005 (ANOVA); Error bars: s.e.m. (**c**–**f**) The putative AKT phosphorylation site T10 in OSM-9 is required for H_2_O_2_-induced potentiation of ASH sensory response. In *osm-9* mutant worms, ASH neurons did not respond to glycerol (**c**), and this defect was rescued by wild-type *osm-9* transgene (**d**). Wild-type *osm-9* transgene also rescued H_2_O_2_-induced potentiation of ASH sensory response (**d**). However, *osm-9* transgene with the point mutation T10A (that is *ASH::osm-9(T10A)*) did not rescue H_2_O_2_-induced potentiation of ASH sensory response, though it rescued the basal glycerol sensory response in ASH neurons(**e**). Shades along the traces in (**c**–**e**) represent error bars (s.e.m.). Bar graph in (**f**) summarizes the data in (**c**–**e**). *n*≥8; Error bars: s.e.m. *P<0.05 (ANOVA).

As OSM-9 is predicted to possess five putative AKT phosphorylation sites and it is also the primary sensory channel in ASH neurons, we hypothesize that OSM-9 may be a downstream target of AKT. To test this idea, we asked whether any of those putative AKT phosphorylation sites in OSM-9 is critical for H_2_O_2_-induced behavioral potentiation (Fig. 6a). Among the five putative AKT sites, one is located at the N-terminal end of OSM-9 while the other four are found at the C-terminus (Fig. 6a). We mutated all four C-terminal AKT sites in *osm-9* cDNA from S/T to A, and introduced this ‘quad mutant' as a transgene in ASH neurons in the *osm-9* mutant background. As reported previously^11^,^31^, *osm-9* mutant worms did not respond to high osmolarity stimulus (Fig. 6b) (note: the basal response rate in the mutant arose from stimulus-independent spontaneous reversal events). As was the case with *osm-9* wild-type transgene, *osm-9(quad mutant)* transgene expressed in ASH neurons can rescue osmotic avoidance behavioral response in naive worms, as well as behavioral potentiation in H_2_O_2_-treated worms (Fig. 6b). We conclude that the four putative AKT sites at the C-terminal end of OSM-9 are not required for H_2_O_2_-induced behavioral potentiation.

We then turned our attention to the putative AKT site at the N-terminus of OSM-9. We generated a transgene *osm-9*(*T10A*) harboring a point mutation with this site changed to A (Fig. 6a). As was the case with wild-type *osm-9* transgene, expression of *osm-9*(*T10A*) transgene in ASH neurons of *osm-9* mutant worms rescued the basal osmotic behavioral response (Fig. 6b). This shows that the basic function of OSM-9 was not compromised by T10A mutation. However, this *osm-9*(*T10A*) transgene failed to rescue H_2_O_2_-induced behavioral potentiation (Fig. 6b), revealing a requirement for the putative phosphorylation site T10 in mediating H_2_O_2_-induced behavioral potentiation.

To gather functional evidence, we conducted calcium imaging experiments. Similar to wild-type *osm-9* transgene (Fig. 6c,d), *osm-9*(*T10A*) transgene also rescued the basal ASH sensory response in *osm-9* mutant worms (Fig. 6e,f), supporting the notion that the basic function of OSM-9 was not compromised by T10A mutation. However, this *osm-9*(*T10A*) transgene failed to rescue H_2_O_2_-induced potentiation of ASH sensory response (Fig. 6e,f). By contrast, *osm-9(quad mutant)* transgene, in which all four C-terminal AKT sites were mutated, rescued H_2_O_2_-induced potentiation of ASH sensory response (Supplementary Fig. 8). This set of experiments uncovers a specific role for the putative AKT phosphorylation site T10 in OSM-9 in mediating H_2_O_2_-induced potentiation of sensory behavior and ASH neuron function.

To provide additional evidence, we recorded OSM-9-mediated sensory current by patch-clamp. Perfusion of glycerol-containing high osmolarity solution evoked an inward current in ASH neurons (Supplementary Fig. 9a,b). H_2_O_2_ treatment potentiated this current (Supplementary Fig. 9a,b), which is consistent with our behavioral and calcium imaging data. Using the same strategy as behavioral analysis and calcium imaging, we introduced transgenes expressing *osm-9* cDNA in ASH neurons in *osm-9* mutant background. Wild-type *osm-9* transgene retained the ability to mediate H_2_O_2_-induced potentiation of sensory current (Supplementary Fig. 9c,d). By contrast, the *osm-9(T10A)* transgene, which encompasses a point mutation disrupting the putative AKT phosphorylation site, lost the ability to mediate H_2_O_2_-induced potentiation of sensory current (Supplementary Fig. 9e,f). This patch-clamp data presents additional evidence supporting a key role for the putative AKT site T10 in OSM-9 in mediating H_2_O_2_-induced potentiation of ASH sensory response.

### AKT-1 phosphorylates OSM-9 at Thr10 *in vitro*

**Figure 7:**
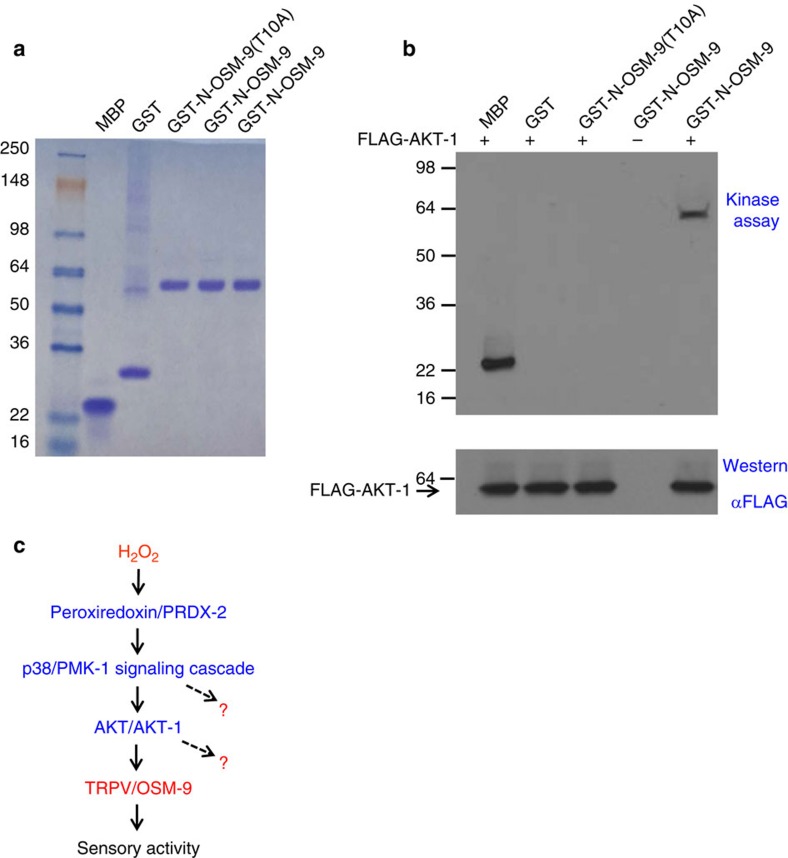
AKT-1 phosphorylates OSM-9 *in vitro*. (**a**) *In vitro* production of GST fusion proteins of OSM-9. The N-terminal fragment of wild-type and point mutant (T10A) of OSM-9 was fused to GST, expressed and purified from bacterial strain BL21. Shown is a SDS-PAGE gel stained by Coomassie blue. The pan-kinase substrate MBP and GST alone were also shown. (**b**) AKT-1 phosphorylates OSM-9 in an *in vitro* kinase assay. FLAG-tagged AKT-1 was transfected in HEK293T cells. After pull-down by anti-FLAG affinity beads, AKT-1 was tested for its ability to phosphorylate purified MBP, GST, and GST fusion proteins using (γ-^32^P)ATP as the substrate. Top panel: autoradiograph. Bottom panel: Western blot showing the amount of AKT-1 used in each experiment. (**c**) A schematic model illustrating the signaling pathway that mediates H_2_O_2_-induced potentiation of neuronal activity. Question marks indicate that additional substrates may exist.

The above data raised the intriguing possibility that the TRPV channel OSM-9 may be a substrate for AKT-1 kinase. To test this model, we performed an *in vitro* kinase assay. We produced the N-terminal fragment of OSM-9 as a GST fusion protein (that is GST-N-OSM-9) and purified it from bacteria lysate (Fig. 7a). The pan-kinase substrate MBP served as a positive control (Fig. 7a,b). Notably, AKT-1 transfected in HEK293 cells can phosphorylate GST-N-OSM-9 but not GST alone or GST-N-OSM-9(T10A) that harbors the point mutation T10A (Fig. 7b). Thus, AKT-1 can phosphorylate OSM-9 *in vitro*, identifying OSM-9 as a substrate for AKT-1.

## Discussion

Owing to their deleterious effects on cellular functions, ROS have been implicated in a large variety of human diseases particularly neurodegenerative diseases^2^,^4^. In the current study, we demonstrate that unlike the commonly observed adverse effects of ROS, low doses of H_2_O_2_ can in fact promote sensory behavior and potentiate sensory neuron function in *C. elegans*, revealing a beneficial role of ROS in the nervous system. We also show that reducing the level of endogenous H_2_O_2_ compromises sensory behavior, suggesting that the role of H_2_O_2_ in promoting neuronal function is physiological. Such an effect of ROS may benefit the survival of *C. elegans*. For example, ROS are often associated with harsh environments. Sensitization of sensory neurons, such as the polymodal neuron ASH, by ROS would render the animals more responsive to noxious cues and thereby facilitate their escape from harsh environments. Meanwhile, as ROS can also promote the activity of chemosensory neurons such as AWA that detects food sources, this effect may also help the animals to approach pleasant environments.

Our results point to a model in which H_2_O_2_ promotes sensory behavior and ASH neuron function through a peroxiredoxin-p38/MAPK signaling axis (Fig. 7c). In this model, we propose that peroxiredoxin acts as a H_2_O_2_ sensor that transduces information through p38/MAPK signaling which in turn signals to AKT and the sensory channel OSM-9/TRPV in ASH neurons (Fig. 7c), and that AKT regulates the OSM-9/TRPV sensory channel by phosphorylating its N-terminus. As all the members in the peroxiredoxin-p38/MAPK signaling pathway are evolutionarily conserved across phylogeny, our results raise the intriguing possibility that H_2_O_2_ may play a similar role in regulating brain function in other organisms.

In addition to their role in the brain, ROS have been proposed to underlie cellular senescence of other cell types and affect organismal aging^36^. Interestingly, recent studies in *C. elegans* show that accumulation of ROS is not causal in the aging process and a mild increase in ROS production in mitochondria of ASI diet-sensitive neurons may even prolong lifespan through mitohormesis^6^,^7^,^36^,^37^. Though the underlying mechanisms are not well understood, p38/PMK-1 and SKN-1/Nrf2 are found to be involved^20^,^37^,^38^. This reveals a common role of p38/PMK-1 in mediating both ROS-mediated extension of lifespan and potentiation of neuronal function. Nevertheless, the signaling events downstream of p38/PMK-1 appear to be different in these two processes.

High doses of H_2_O_2_ compromise sensory behavior and sensory neuron function. One possibility is that this deleterious effect of H_2_O_2_ may result from its non-specific modification of proteins, nucleic acids and lipids. Interestingly, we find that this effect can also be blocked by loss of PRDX-2, indicating that it involves specific genes. On the other hand, mutations in p38/PMK-1 and AKT-1 cannot block this detrimental effect. Thus, other signaling pathways are probably also involved. We suggest that under low concentrations, ROS may primarily function as signaling molecules regulating specific cellular pathways, the outcome of which could be beneficial, while under high doses, ROS would become harmful by acting as signaling molecules and also by non-specifically modifying proteins, lipids and nucleic acids.

We identify the TRPV channel OSM-9 as a critical substrate for the peroxidoxin-p38/MAPK signaling pathway in ASH sensory neurons. Some TRP channels such as TRPA, TRPV and TRPC channels can be directly regulated by ROS through oxidation^39^. We thus do not rule out the possibility that OSM-9 may also be regulated by this mechanism. It should be noted that OSM-9 is probably not the only target of the peroxidoxin-p38/MAPK signaling pathway in ASH neurons. In other words, this signaling cascade may impinge on additional substrates in ASH, but the identities of which remain to be identified (Fig. 7c). These additional substrates may contribute to H_2_O_2_-induced potentiation of sensory behavior and ASH neuron function. It is also possible that peroxiredoxin-p38/MAPK signaling may recruit different sets of substrates in different classes of neurons. Lastly, we do not rule out the possibility that pathways other than peroxiredoxin-p38/MAPK may contribute to the observed beneficial effect of H_2_O_2_. Future effort is needed to address these intriguing questions. The current study unveils unexpected complexity of the action of ROS in the nervous system, providing an entry point to understanding the multifaceted roles of ROS in health and disease.

## Methods

### Strains

See Supplementary Table 1.

### Behavioral assays

Osmotic avoidance behavior was carried out at 20 °C using a drop test assay as described previously^40^,^41^. Day 1 adult hermaphrodites were tested. We placed a small drop of glycerol-containing solution (M9) in the path of a worm moving forward. A positive response was scored if the worm stopped forward movement and also initiated a reversal that lasted at least half a head swing. The assay was conducted on NGM plates without bacteria. To avoid a ceiling effect that would mask behavioral potentiation, we used a non-saturating concentration of glycerol (for example 0.25–0.5 M) unless otherwise specified. Each worm was tested five times and a response rate was calculated for each animal. In general, 10–20 worms were assayed for each experiment (*n* number=10–20 worms), which should be sufficient based on power analysis. Chemotaxis assay was performed using the standard protocol described previously^15^. To avoid a ceiling effect, we used a non-saturating concentration of diacetyl (50,000 dilution) unless otherwise indicated.

To treat worms with H_2_O_2_, we incubated them with varying concentrations of H_2_O_2_ diluted in M9 buffer for 2 h. Fresh OP50 bacteria were included in M9 buffer to prevent starvation during H_2_O_2_ treatment. After treatment, worms were allowed to recover on seeded NGM plates for up to 2 h before behavioral, imaging and patch-clamp analysis. Mock-treated worms were used as a control. We also tested untreated worms (left on seeded NGM plates for 2 h) and found no difference from mock-treated worms (Supplementary Fig. 1). To test whether reducing the level of endogenous H_2_O_2_ would affect osmotic avoidance behavior, we treated worms with BHA or NAC for 2 h (OP50 included) and then assayed worms right after the treatment, since recovering worms on NGM plates would allow BHA and NAC to diffuse out, which would presumably lead to a rebound in H_2_O_2_ level inside the cell. 0.1 μM H_2_O_2_ was used to induce potentiation of osmotic avoidance behavior and ASH neuron sensory response throughout the paper unless otherwise indicated.

### Calcium imaging

A microfluidic system was used to perform calcium imaging on Day 1 adult worms^42^. Worms were loaded onto a microfluidic chip mounted on an upright microscope (Olympus BX51WI), and incubated in standard bath solution (in mM): 145 NaCl, 2.5 KCl, 1 CaCl_2_, 1 MgCl_2_, 20 glucose and 5 HEPES (320 mOsm; pH adjusted to 7.3). The images were acquired with a Roper Coolsnap CCD camera and analysed with MetaFluor software (Molecular Devices, Inc.). GCaMP6 and DsRed were co-expressed as a transgene in ASH under the *sra-6* promoter to enable ratiometric imaging using blue and red light^43^,^44^,^45^. As ASH neurons are photosensitive^46^, we first exposed them to blue light for 2 min to bleach their intrinsic photosensitivity before applying chemical stimuli. The peak fold change in the ratio of G-CaMP/DsRed fluorescence was analysed. Each worm was only imaged once.

### Electrophysiology

Whole-cell recordings were performed on an upright microscope (BX51WI) with an EPC-10 amplifier^45^,^47^,^48^. Worms were glued and dissected on a sylgard-coated coverglass to expose ASH neurons. Bath solution (in mM): 145 NaCl, 2.5 KCl, 1 CaCl_2_, 1 MgCl_2_, 20 glucose and 5 HEPES (320 mOsm; pH adjusted to 7.3). Pipette solution: 115 K-gluconate, 15 KCl, 1 MgCl_2_, 10 HEPES, 0.25 CaCl_2_, 20 sucrose, 5 BAPTA, 5 Na_2_ATP and 0.5 NaGTP (315 mOsm; pH adjusted to 7.2). DsRed and YFP fluorescence markers were expressed as a transgene (*xuEx631*) to label and identify ASH neurons for recording. Clamping voltage: −60 mV. Series resistance and membrane capacitance were both compensated during recording.

### Biochemistry

GST fusion proteins were expressed and purified from BL21 bacteria using the standard protocol^49^. Briefly, after IPTG induction (1 mM, 5 h), bacteria were pelleted and resuspended in TEN buffer (10 mM Tris, pH 8.0, 1 mM EDTA, 0.4% NP-40 and 1 mM DTT), and lysed by sonication. GST fusions were then purified using glutathione beads. To prepare for kinase assay, worm FLAG AKT-1 was cloned into pcDNA3 vector and transfected into HEK293T cells. Cells were lysed with lysis buffer (100 mM Tris pH 7.5, 1% NP-40, 10% glycerol, 130 mM sodium chloride, 5 mM MgCl_2_, 1 mM sodium vanadate, 1 mM NaF and 1 mM EDTA) supplemented with protease inhibitor cocktail (Roche). FLAG-AKT-1 was then pulled down by immunoprecipitation with anti-FLAG affinity beads. *In vitro* kinase assay was performed by incubating GST fusion proteins and FLAG-AKT-1-bound affinity beads in 50 μl kinase buffer (50 mM MOPS pH 7.2, 50 mM NaCl, 25 mM b-glycerolphosphate, 10 mM MgCl_2_, 1 mM sodium vanadate, 1 mM NaCl and 100 uM ATP). 5 μCi of (γ^32^P)ATP and lipid activator mix (Millipore) were included in the kinase reaction. The reaction was stopped by adding 5 × SDS–polyacrylamide gel electrophoresis (SDS–PAGE) sample buffer, and the proteins were separated by SDS–PAGE and transferred to nitrocellulose membrane followed by autoradiography and Western probed with an anti-FLAG antibody (1:5,000 dilution, Cat#. F4042, Sigma). The Western image in Supplementary Fig. 7b was cropped for presentation, and the full size image is presented in Supplementary Fig. 10.

### Data Availability

All custom strains have been made available at http://cbs.umn.edu/cgc/home or http://shigen.nig.ac.jp/c.elegans/. Additional modified strains can be accessed upon request. The data that support the findings of this study are available from the corresponding author on reasonable request.

## Additional information

**How to cite this article**: Li, G. *et al*. Promotion of behavior and neuronal function by reactive oxygen species in *C. elegans*. *Nat. Commun.* **7**, 13234 doi: 10.1038/ncomms13234 (2016).

**Publisher's note:** Springer Nature remains neutral with regard to jurisdictional claims in published maps and institutional affiliations.

## Supplementary Material

Supplementary Information Supplementary Figures 1-10 and Supplementary Table 1.
