# Supplementary material for: Promotion of behavior and neuronal function by reactive oxygen species in C. elegans
Source: Nat Commun. 2016 Nov 8;7:13234. doi: 10.1038/ncomms13234 (PMC5105148; doi:10.1038/ncomms13234)
Supplement: Supplementary Information — Supplementary Figures 1-10 and Supplementary Table 1. [file ncomms13234-s1.pdf]

Supplementary Figure 1

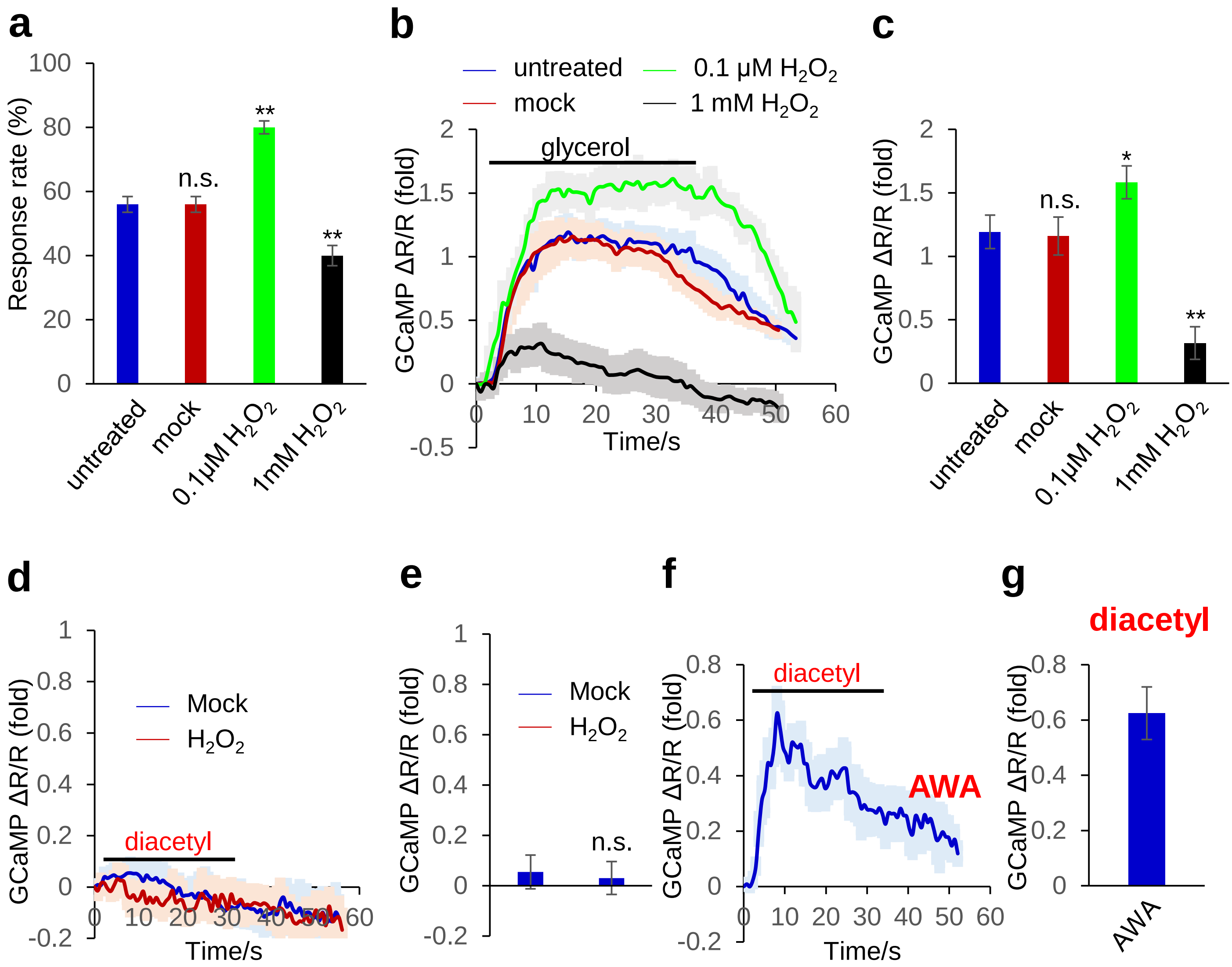

Supplementary Figure 1. Additional controls related to H_2O_2-indcued potentiation and suppression of osmotic avoidance behavior and ASH neuron response.

(a) Untreated worms and mock-treated worms showed no notable difference in osmotic avoidance behavior. Untreated: worms were left untreated on seeded NGM plates for the same duration of time. Mock: worms were mock-treated with M9 buffer (OP50 bacteria included). **$p<0.005$ (ANOVA with Dunnett's test, all compared to untreated worms). n=20. Error bars: SEM.

(b-c) Untreated worms and mock-treated worms showed no notable difference in ASH sensory response. (b) sample traces. (c) bar graph. n≥10; *$p<0.05$, **$p<0.005$ (ANOVA with Dunnett's test, all compared to untreated worms); Error bars: SEM.

(d-e) ASH neurons remain unresponsive to diacetyl after H_2O_2 (0.1 μM) treatment. ASH neurons were tested for diacetyl (1:10,000) sensitivity by calcium imaging. (d) sample traces. (e) bar graph. n=8. Error bars: SEM.

(f-g) AWA neurons respond to diacetyl. As a positive for (d-e), we found that diacetyl can evoke calcium transients in AWA neurons. n=9. Error bars: SEM.

Supplementary Figure 2

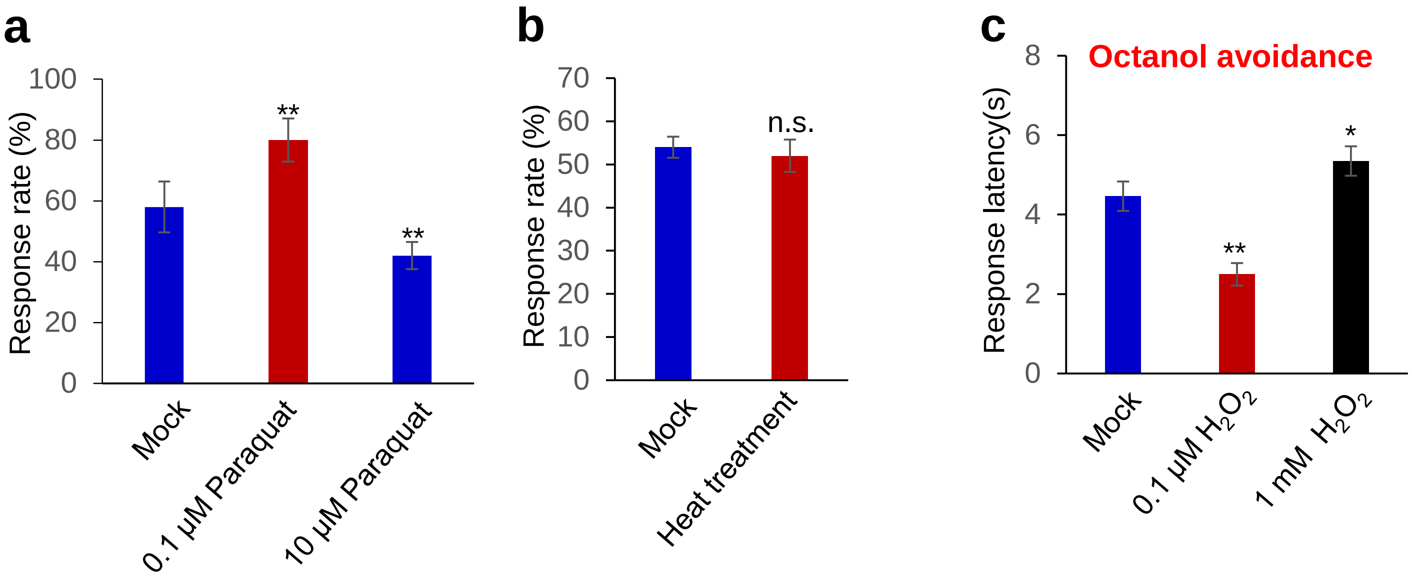

Supplementary Figure 2. The effect of paraquat and heat treatment on osmotic avoidance behavior, and the effect of H_2O_2 treatment on octanol avoidance behavior.
(a) Paraquat treatment potentiates and suppresses osmotic avoidance behavior, depending on its concentration. The treatment protocol is the same as that used for H_2O_2. $^{**}p<0.005$ (ANOVA with Dunnett's test). n=10. Error bars: SEM.
(b) Heat treatment does not elicit a notable change in osmotic avoidance behavior. The treatment protocol is similar to that used for H_2O_2, except that worms were treated with heat (30 ^{o}C for 2 hours). n=10. Error bars: SEM.
(c) H_2O_2 treatment potentiates and suppresses octanol avoidance behavior, depending on its concentration. To assay octanol avoidance behavior, octanol odor (20%) was presented to a forward-moving worm using a mouse pipette. The latency time that it took a worm to initiate reversals was quantified. The shorter the latency, the more robust the behavioral response is. $n \geq 14$. $^{*}p<0.05$, $^{**}p<0.005$ (ANOVA with Dunnett's test). Error bars: SEM.

Supplementary Figure 3

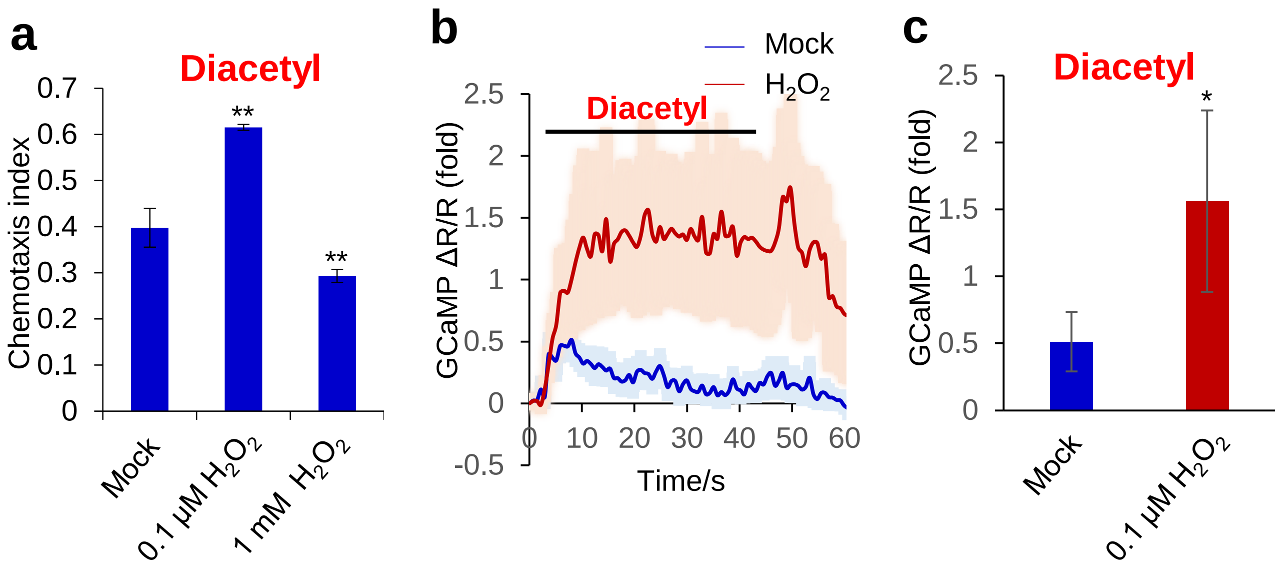

Supplementary Figure 3 H_2O_2 treatment promotes olfactory behavior and olfactory neuron function.
(a) Low doses of H_2O_2 promote olfactory behavior while high doses inhibit it. Worms were pre-incubated with low (0.1 μM) and high concentration (1 mM) of H_2O_2 for 2 hours prior to testing chemotaxis behavior in response to diacetyl. To avoid a ceiling effect which would mask behavioral potentiation, a non-saturating concentration of diacetyl (1:50000 dilution) was used. **p<0.005, (ANOVA with Dunnett's test). n≥5. Error bars: SEM.
(b-c) H_2O_2 treatment potentiates the sensory response of AWA neurons. To enable ratiometric calcium imaging, GCaMP6 and DsRed were co-expressed in AWA neurons as a transgene using the *srx-47* promoter. Worms were pre-treated with H_2O_2 (0.1 μM) for 2 hours, and AWA neurons were recorded for their response to diacetyl (1:100000 dilution). Shades along the calcium traces in (b) represent error bars (SEM). Bar graphs in (c) summarizing the data in (b). n≥8; *p<0.05 (ANOVA test); Error bars: SEM.

Supplementary Figure 4

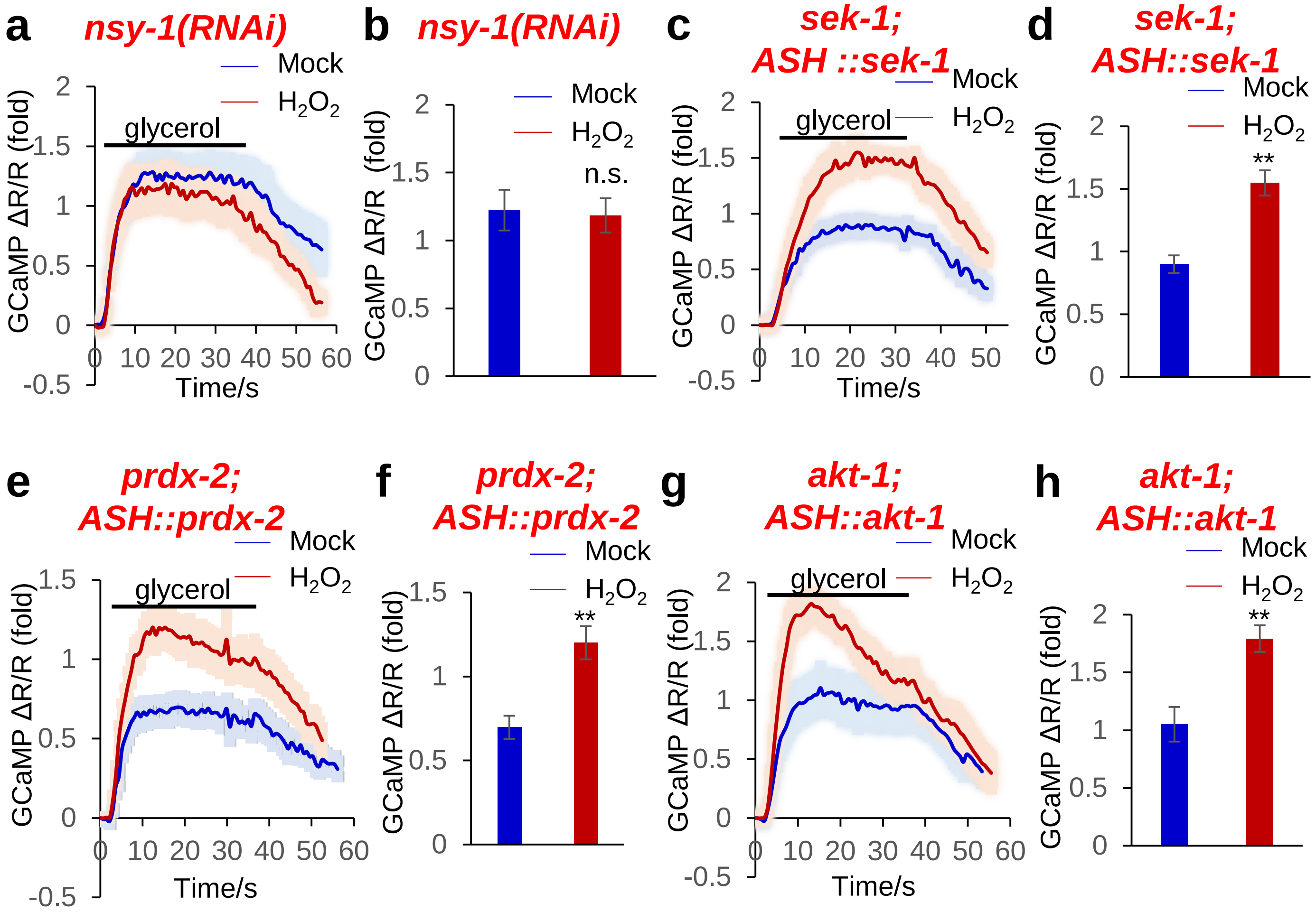

Supplementary Figure 4. Neuron-specific RNAi and rescuing data related to some of the genes in the peroxiredoxin-p38/MAPK pathway.
(a-b) NSY-1 acts in ASH neurons to mediate H_2O_2-induced potentiation of ASH sensory response. RNAi of *nsy-1* gene in ASH neurons of wild-type worms abolished the ability of H_2O_2 to promote ASH calcium response to glycerol. *nsy-1* RNAi was expressed as a transgene in wild-type worms using the *sra-6* promoter. Shades along the traces in (a) represent error bars (SEM). Bar graph in (b) summarizes the data in (a). n≥9; Error bars: SEM.
(c-d) SEK-1 acts in ASH neurons to mediate H_2O_2-induced potentiation of ASH sensory response. Transgenic expression of *sek-1* cDNA in ASH neurons of *sek-1* mutant worms using the *sra-6* promoter rescued the ability of H_2O_2 to promote ASH sensory response. Shades along the calcium traces in (c) represent error bars (SEM). Bar graphs in (d) summarizing the data in (c). n≥12; **p<0.005 (ANOVA test); Error bars: SEM.
(e-f) PRDX-2 acts in ASH neurons to mediate H_2O_2-induced potentiation of ASH sensory response. Transgenic expression of *prdx-2* cDNA in ASH neurons of *prdx-2* mutant worms using the *sra-6* promoter rescued the ability of H_2O_2 to promote ASH sensory response. Shades along the calcium traces in (e) represent error bars (SEM). Bar graphs in (f) summarizing the data in (e). n≥12; **p<0.005 (ANOVA test); Error bars: SEM.
(g-h) AKT-1 acts in ASH neurons to mediate H_2O_2-induced potentiation of ASH sensory response. Transgenic expression of *akt-1* cDNA in ASH neurons of *akt-1* mutant worms using the *sra-6* promoter rescued the ability of H_2O_2 to promote ASH sensory response. Shades along the calcium traces in (g) represent error bars (SEM). Bar graphs in (h) summarizing the data in (g). n≥10; **p<0.005 (ANOVA test); Error bars: SEM.

Supplementary Figure 5

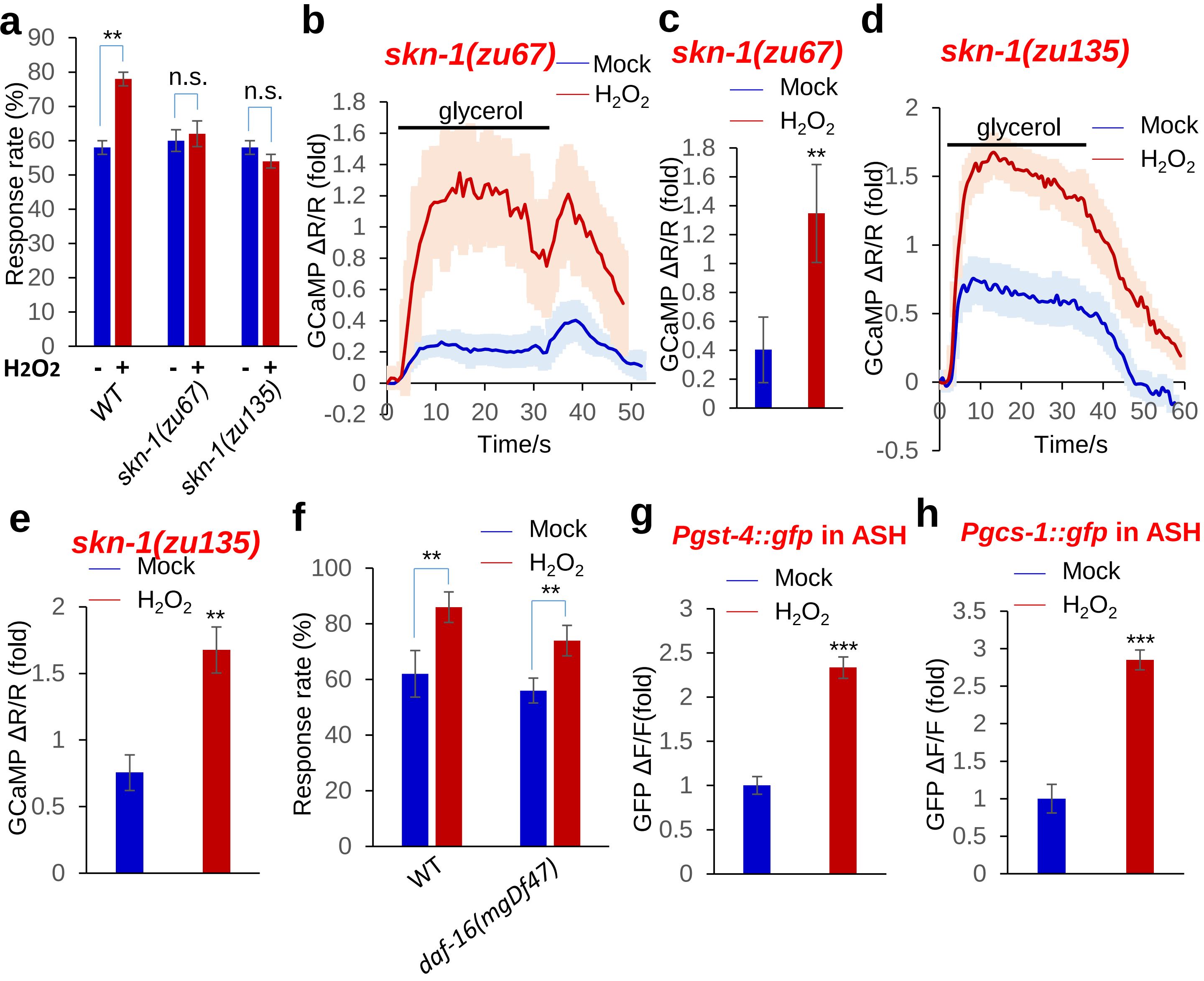

Supplementary Figure 5. SKN-1 is not required for H_2O_2-induced potentiation of ASH neuron sensory response.
(a) SKN-1 is required for H_2O_2-induced behavioral potentiation. H_2O_2 treatment failed to promote osmotic avoidance behavior in *skn-1(zu67)* and *skn-1(zu135)* mutant worms. n≥20; **p<0.005 (ANOVA test); Error bars: SEM.
(b-e) SKN-1 is not required for H_2O_2-induced potentiation of ASH neuron sensory response. Calcium imaging shows that H_2O_2 treatment failed to promote ASH sensory response in *skn-1(zu67)* and *skn-1(zu135)* mutant worms Shades along the calcium traces in (b) and (d) represent error bars (SEM). Bar graphs in (c) and (e) summarize the data in (b) and (d), respectively. n=8; **p<0.005 (ANOVA test); Error bars: SEM.
(f) DAF-16 is not required for H_2O_2-induced behavioral potentiation. n=10, **p<0.005 (ANOVA test), Error bars: SEM.
(g-h) H_2O_2 treatment stimulates the expression of *gst-4* and *gcs-1* genes in ASH neurons. ASH neurons were identified by another transgene expressing DsRed in ASH under the *sra-6* promoter. Both *gst-4* and *gcs-1* were found to be expressed in ASH neurons. Worms were treated with 0.1 μM H_2O_2 for 2 hours before imaging analysis, and the images were quantified with ImageJ. n≥12; ***p<0.0005 (ANOVA test); Error bars: SEM.

Supplementary Figure 6

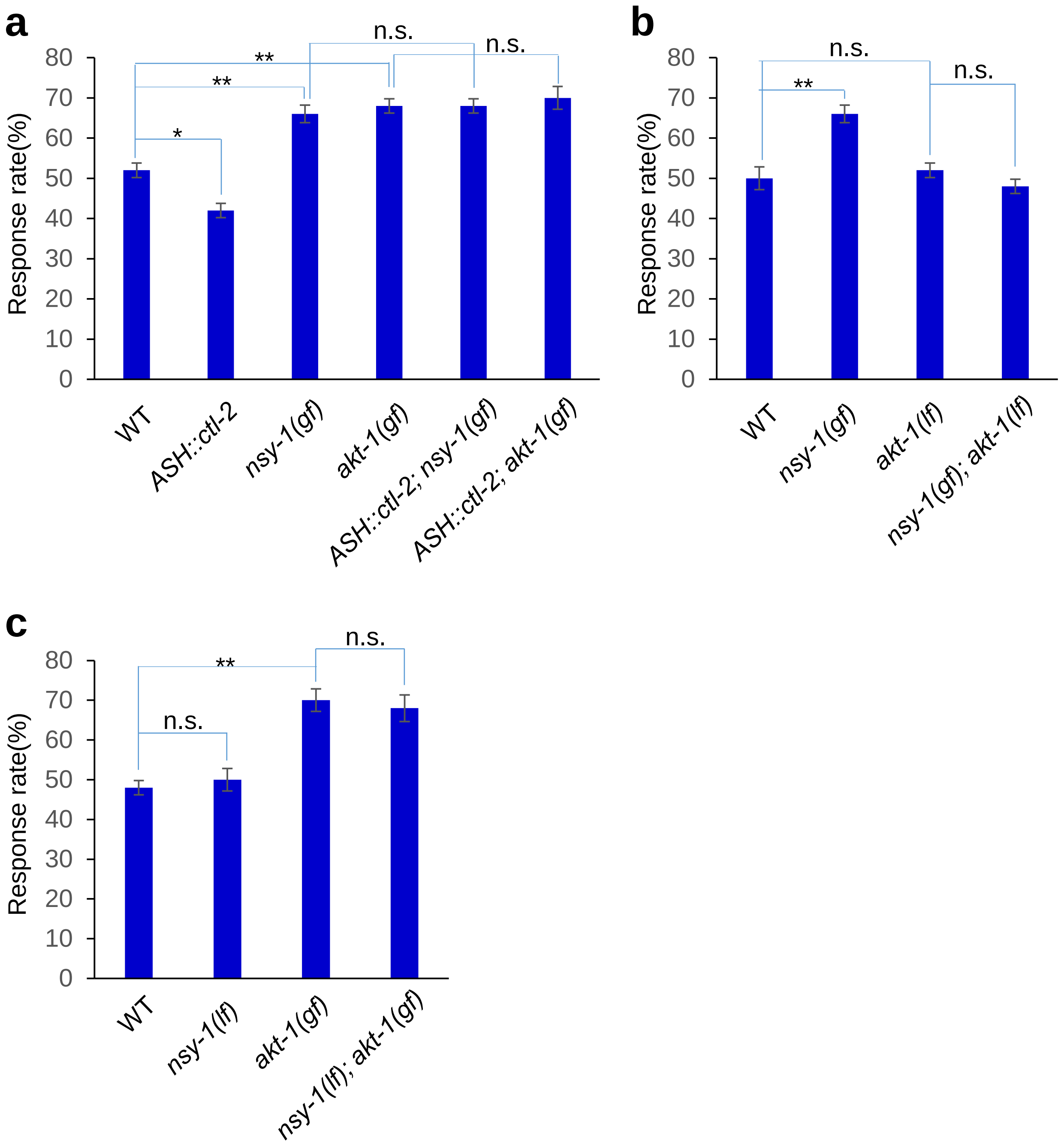

Supplementary Figure 6. Epistatic analysis.
(a) *nsy-1* and *akt-1* act downstream of H_2O_2. *ASH::ctl-1* refers to worms expressing a catalase (*ctl-2*) transgene specifically in ASH neuron. *nsy-1(gf)* and *nsy-1(lf)* refers to *nsy-1* gain-of-function and loss-of-function allele *ums8* and *ok593*, respectively. *akt-1(gf)* and *akt-1(lf)* refers to *akt-1* gain-of-function and loss-of-function allele *mg144* and *mg306*, respectively. n=10, *p<0.05, **p<0.005 (ANOVA with Tukey test), Error bars: SEM.
(b-c) *akt-1* acts downstream of *nsy-1.* n=10, **p<0.005 (ANOVA with Tukey test), Error bars: SEM.

Supplementary Figure 7

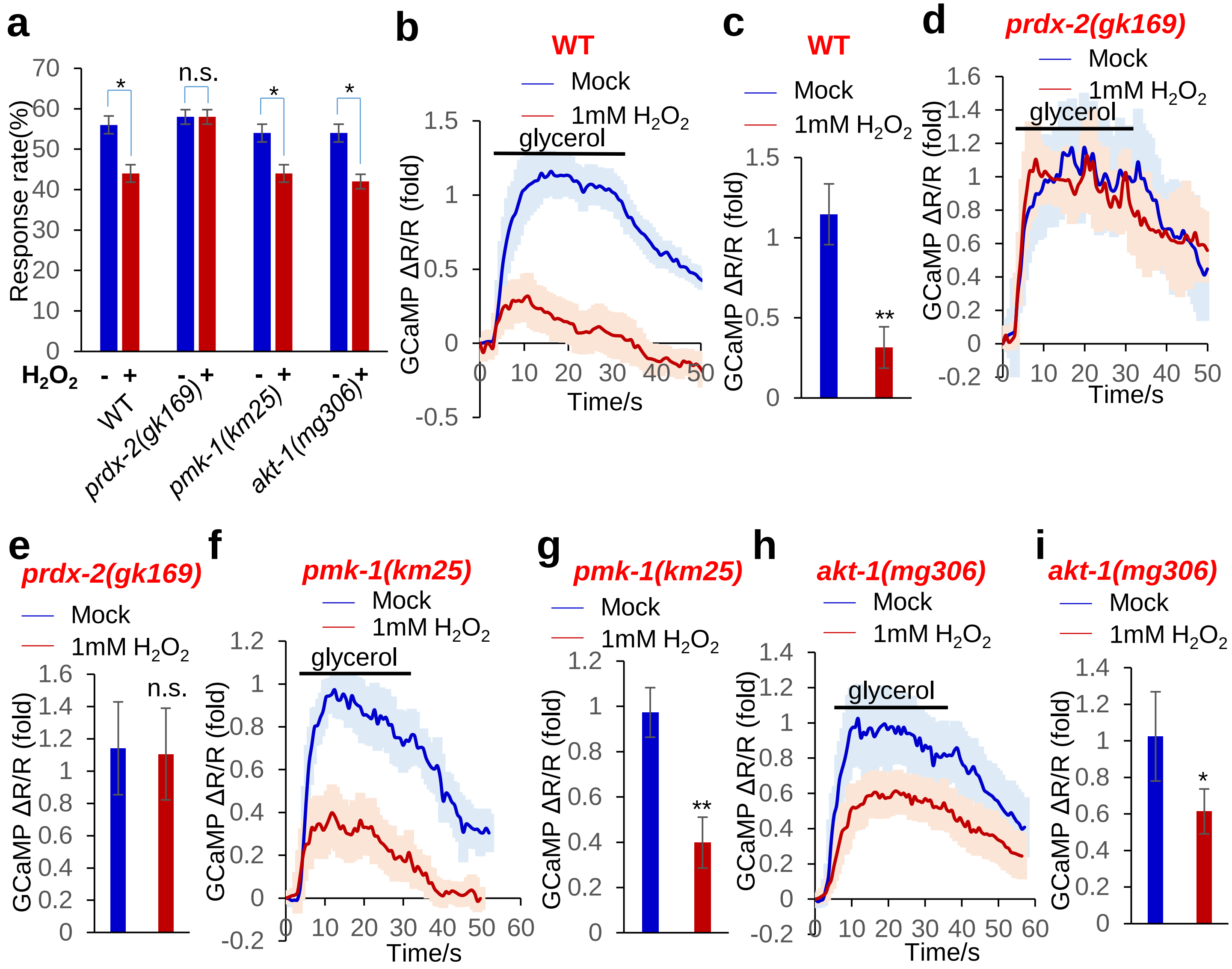

Supplementary Figure 7. High doses of H_2O_2-induced suppression of osmotic avoidance behavior and ASH sensory response requires PRDX-2 but not p38/PMK-1 or AKT-1.

(a) High doses of H_2O_2-induced suppression of osmotic avoidance behavior requires PRDX-2 but not p38/PMK-1 or AKT-1. n=20, *p<0.05 (ANOVA test), Error bars: SEM.

(b-c) High doses of H_2O_2 suppresses ASH sensory response. (b) calcium imaging traces. (c) bar graph. n≥10, **p<0.005 (ANOVA test), Error bars: SEM.

(d-e) High doses of H_2O_2-induced suppression of ASH sensory response requires PRDX-2 n=8, ANOVA test, Error bars: SEM.

(f-g) High doses of H_2O_2-induced suppression of ASH sensory response does not require PMK-1. n=9, **p<0.005 (ANOVA test), Error bars: SEM.

(h-i) High doses of H_2O_2-induced suppression of ASH sensory response does not require AKT-1. n≥8, *p<0.05 (ANOVA test), Error bars: SEM.

Supplementary Figure 8

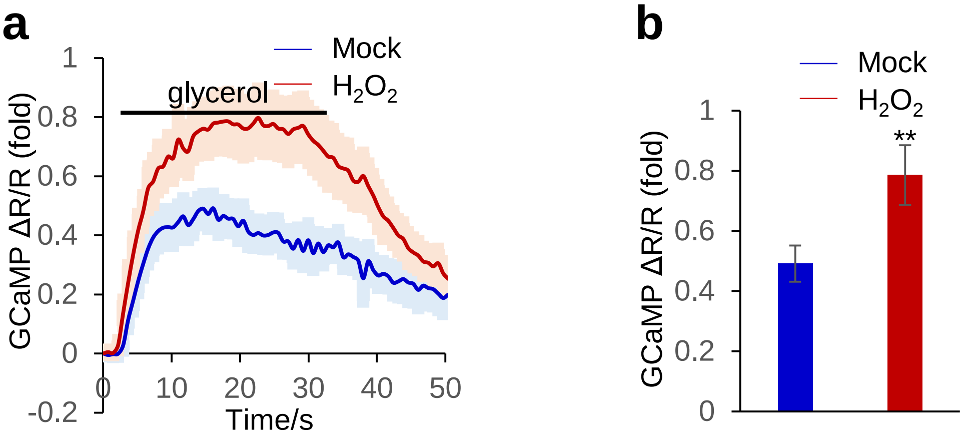

Supplementary Figure 8. *osm-9(quad mutant)* transgene, in which all four C-terminal AKT sites are mutated, can rescue H_2O_2-induced potentiation of ASH sensory response. Genotype: *osm-9(ky10); ASH::osm-9(quad mutant)*. **(a)** Calcium imaging traces. Shades along the traces denote error bars (SEM). **(b)** Bar graph. n=8, **$p<0.005$ (ANOVA test). Error bar: SEM. The basal calcium responses to glycerol in these worms seemed to be a bit lower than other transgenes. This may be caused by the low expression level of this mutant transgene; alternatively, the quadruple point mutations might have slightly compromised the channel function. Regardless, these four putative AKT sites are not required for mediating H_2O_2-induced potentiation of ASH sensory response.

Supplementary Figure 9

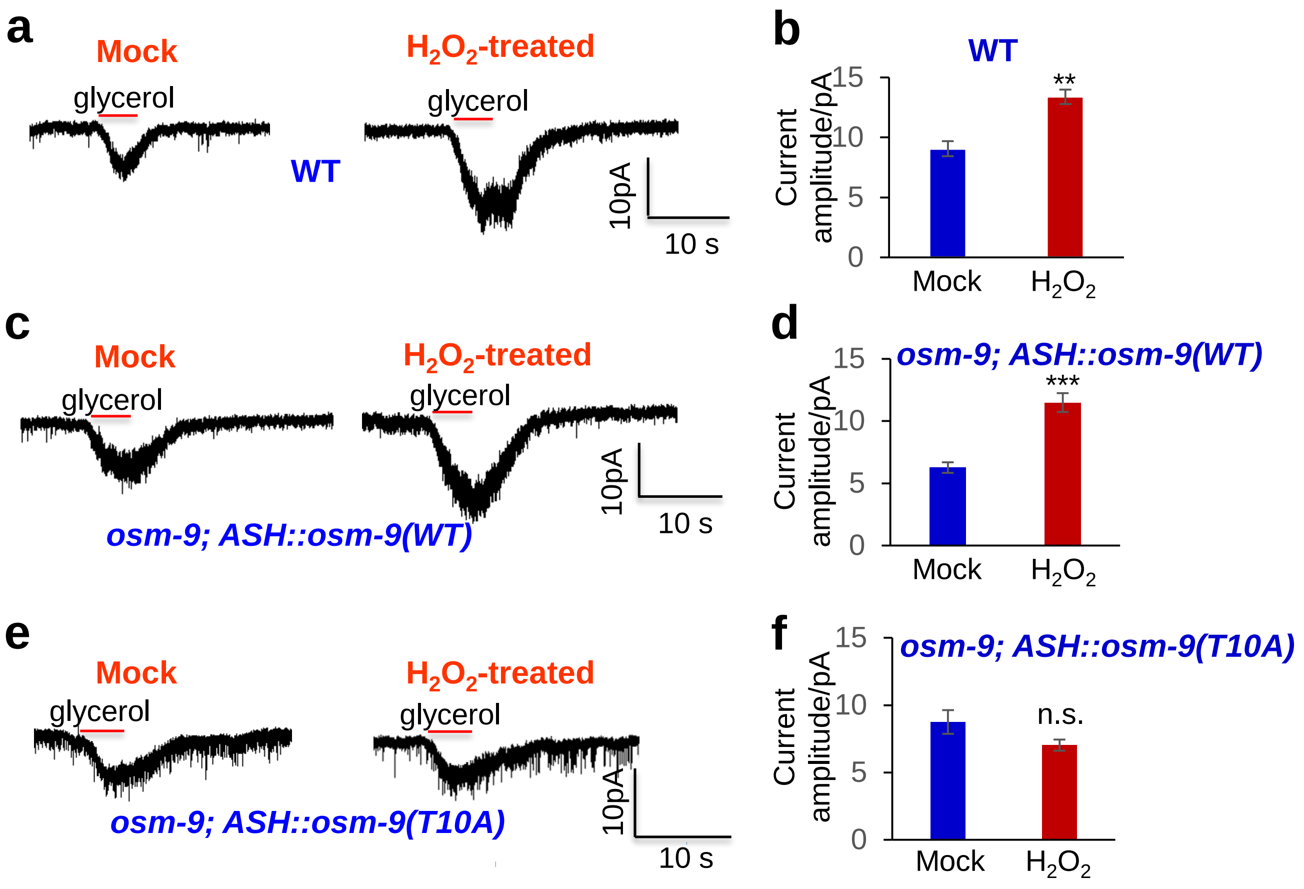

Supplementary Figure 9. The putative AKT phosphorylation site T10 in OSM-9 is required for H_2O_2-induced potentiation of ASH sensory response.
(a-b) H_2O_2 treatment potentiates glycerol-evoked electric current in ASH neurons. Worms were pre-treated with H_2O_2 (0.1 µM) for 2 hours, and ASH neurons were recorded for their response to glycerol under voltage clamp. To avoid a ceiling effect, a non-saturating concentration of glycerol (0.25M) was used to evoke the current. Voltage: -60 mV. Bar graphs in (b) summarizing the data in (a). $n \geq 9$; $^{**}p<0.0005$ (ANOVA test); Error bars: SEM.
(c-d) Wild-type OSM-9 retains the ability to mediate of H_2O_2-induced potentiation of sensory current in ASH neurons. Wild-type *osm-9* cDNA was expressed as a transgene in ASH neurons under the *sra-6* promoter in *osm-9* mutant worms. Voltage: -60 mV. Bar graphs in (d) summarizing the data in (c). $n \geq 9$; $^{**}p<0.0005$ (ANOVA test); Error bars: SEM.
(e-f) The putative AKT phosphorylation site T10 in OSM-9 is required for H_2O_2-induced potentiation of sensory current in ASH neurons. *osm-9* cDNA harboring T10A mutation was expressed as a transgene in ASH neurons under the *sra-6* promoter in *osm-9* mutant worms. Voltage: -60 mV. Bar graphs in (f) summarizing the data in (e). $n \geq 10$; Error bars: SEM. ANOVA test

Supplementary Figure 10

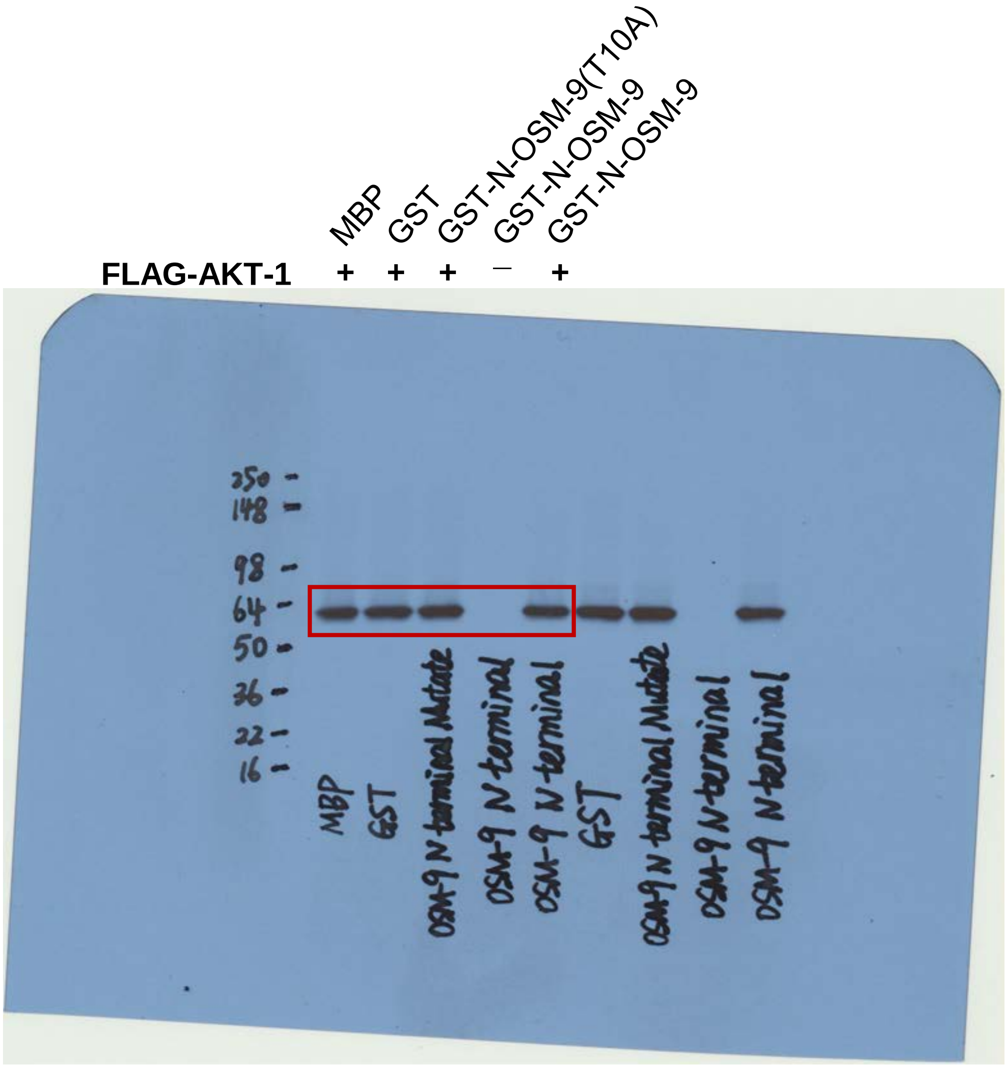

Supplementary Figure 10. The full size image of the Western blot shown in Figure 7b. The lanes in the red box was cropped and shown in Figure 7b. The molecular weight markers were shown to the left.

Supplementary Table 1

strain	genotype
Wild type	N2
TQ7202	*xuEx2644 [Psra-6::ctl-2(cDNA)::sl2::cfp+Punc-122::rfp]*
TQ5856	*xuEx1978 [Psra-6::GCaMP6+Psra-6::sl2::DsRed]*
TQ5633	*pmk-1(km25)*
TQ6135	*xuEx2088 [Psra-6::pmk-1::yfp2];pmk-1(km25)*
TQ5634	*pmk-3(ok169)*
TQ7221	*mpk-1(tm3476)*
TQ7222	*mpk-2(tm3859)*
TQ2170	*jnk-1(gk7)*
TQ5859	*pmk-1(km25); xuEx1978*
TQ7226	*xuEx1978; xuEx2615 [Psra-6::pmk-1(cDNA)::sl2::YFP]; pmk-1(km5)*
TQ5710	*sek-1(km4)*
TQ5711	*nsy-1(ok593)*
TQ6001	*prdx-2(gk169)*
TQ7141	*xuEx2605 [Psra-6::sek-1(cDNA)::sl2::CFP]; sek-1(km4)*
TQ7152	*xuEx2611 [Psra-6::nsy-1(s+as)+Psra-6::sl2::CFP];N2*
TQ7148	*xuEx2609 [Psra-6::prdx-2(cDNA)::sl2::CFP]; prdx-2(gk169)*
TQ6002	*prdx-3(gk529)*
TQ5912	*sek-1(km4); xuEx1978*
TQ7143	*xuEx2605; xuEx1978; sek-1(km4)*
TQ5913	*nsy-1(ok593); xuEx1978*
TQ7157	*xuEx2611; xuEx1978; N2*
TQ6772	*prdx-2(gk169); xuEx1978*
TQ7150	*xuEx2609; xuEx1978; prdx-2(gk169)*
TQ2172	*akt-1(mg306)*
TQ2619	*xuEx2619 [Psra-6::akt-1(cDNA)::sl2::CFP]; akt-1(mg306)*
TQ2927	*akt-2(ok391)*
TQ6400	*akt-1(mg306); xuEx1978*
TQ85	*osm-9(ky10)*
TQ7166	*xuEx2619; xuEx1978; akt-1(mg306)*
TQ6401	*akt-2(ok391); xuEx1978*
TQ6356	*xuEx2205 [Psra-6::osm-9(cDNA)::sl2::CFP]; osm-9(ky10)*
TQ6610	*xuEx2397 [Psra-6::osm-9(T10A)::sl2::CFP]; osm-9(ky10)*
TQ7170	*xuEx2624 [Psra-6::osm-9(T769A,T771A,T787A,S839A)::sl2::CFP]; osm-9(ky10)*
TQ6021	*osm-9(ky10); xuEx1978*
TQ6865	*xuEx1978; xuEx2205; osm-9(ky10)*
TQ6689	*xuEx1978; xuEx2397; osm-9(ky10)*
TQ1764	*xuEx631 [Psra-6::DsRed + Pstr-3::yfp2]*
TQ5664	*osm-9(ky10); xuEx631*
TQ6835	*xuEx631; xuEx2205; osm-9(ky10)*
TQ6688	*xuEx631; xuEx2397; osm-9(ky10)*
TQ7206	*xuEx2644; xuEx1978*
TQ6376	*xuEx2225 [Psrx-47::GCaMP6+ Psrx-47::DsRed]*
TQ3045	*skn-1(zu67); nT1[unc-?(n754dm) let-?]*
TQ3046	*skn-1(zu135); nT1[unc-?(n754dm) let-?]*
TQ5975	*skn-1(zu67); xuEx1978*
TQ7174	*dvIs19[gst-4p::GFP::NLS]; xuEx331[Psra-6::DsRed]*
TQ7187	*xuEx2629 [Podr-1::GCaMP6+ Pstr-2::DsRed]*
TQ7466	*skn-1(zu135); xuEx1978*
TQ7531	*xuEx2852 [Pgcs-1::NLS::GFP+ Psra-6::DsRed]*
TQ7464	*nsy-1(ums8)*
TQ7509	*akt-1(mg144)*
TQ7483	*nsy-1(ums8); xuEx2644 [Psra-6::ctl-2(cDNA)::sl2::cfp+Punc-122::rfp]*
TQ7468	*akt-1(mg144); xuEx2644 [Psra-6::ctl-2(cDNA)::sl2::cfp+Punc-122::rfp]*
TQ7484	*nsy-1(ums8); akt-1(mg306)*
TQ7506	*nsy-1(ok593); akt-1(mg144)*
TQ6802	*xuEx2478 [Psra-6::osm-9(T10E)::sl2::CFP]; osm-9(ky10)*
